# Immune cell–specific SMN expression and sustained immune alterations in treated spinal muscular atrophy

**DOI:** 10.1172/JCI203540

**Published:** 2026-08-25

**Authors:** Ines Tapken, Katharina Rahmel-Stein, Christine Ehlers, Federica Cieri, Nora T. Detering, Tobias Schüning, Bogdan Bjelica, Charlotte Mindermann, Svenja Neuhoff, Linda-Isabell Schmitt, Markus Leo, Tim Hagenacker, Sabine Illsinger, Elia Di Schiavi, Theresa Graalmann, Susanne Petri, Ulrich Kalinke, Peter Claus

**Affiliations:** 1 TWINCORE–Centre for Experimental and Clinical Infection Research, a joint venture between the Hannover Medical School and the Helmholtz Centre for Infection Research, Hannover, Germany.; 2 Centre for Individualised Infection Medicine (CiiM), a joint venture between the Helmholtz Centre for Infection Research and Hannover Medical School, Hannover, Germany.; 3 Department of Gastroenterology, Hepatology, Infectious Diseases and Endocrinology, and; 4 Cluster of Excellence RESIST (EXC 2155), Hannover Medical School, Hannover, Germany.; 5 Hannover Biomedical Research School (HBRS) and Center for Infection Biology (ZIB), Hannover, Germany.; 6 Biomedical Research in End-Stage and Obstructive Lung Disease Hannover (BREATH), German Center for Lung Research (DZL), Hannover, Germany.; 7 Institute of Biosciences and BioResources, National Research Council (CNR), Naples, Italy.; 8 Department of Biology, University of Naples Federico II, Naples, Italy.; 9 Center for Systems Neuroscience (ZSN), Hannover, Germany.; 10 PRACTIS Clinician Scientist Program, Dean’s Office for Academic Career Development, and; 11 Department of Neurology and Clinical Neurophysiology, Hannover Medical School, Hannover, Germany; 12 Clinic for Pediatric Kidney, Liver, Metabolic, and Neurological Diseases, Hannover Medical School, Hannover, Germany.; 13 Department of Neurology, Center for Translational Neuro- and Behavioral Sciences (C-TNBS), University Medicine Essen, Essen, Germany.; 14 Department of Rheumatology and Immunology, Hannover Medical School, Hannover, Germany.; 15 DIAKOVERE Henriettenstift, Clinic for Neurology and Clinical Neurophysiology, Hannover, Germany.; 16 Laboratory of Molecular Neurosciences, Department of Psychiatry, Social Psychiatry and Psychotherapy, Hannover Medical School, Hannover, Germany.

**Keywords:** Immunology, Muscle biology, Neuroscience, Monogenic diseases, Neurological disorders, Neuromuscular disease

## Abstract

Mutations in the survival of motor neuron 1 (*SMN1*) can reduce functional SMN protein levels, which causes spinal muscular atrophy (SMA), a disease affecting the nervous system and peripheral tissues, including the immune system. Yet, SMN expression across immune cell subsets and the effect of SMN-modulating therapies on the immune system remain underexplored. We found that in neonatal mouse spleens, SMN expression was highest in B cells, and was massively reduced in SMA mice. In human PBMCs from adults, DCs and monocytes expressed the highest SMN levels, whereas patients with SMA showed reduced DC frequencies and increased B cell frequencies. Patients receiving systemic versus CNS-restricted therapy showed similar differences in immune cell composition and SMN expression levels. Similarly, an exploratory cohort including untreated patients did not show a substantial treatment-specific effect relative to controls. To assess the effect of differentiation on SMN, we analyzed PMA-treated THP-1 cells and found enhanced aberrant SMN splicing and increased SMN^+^ Cajal bodies. SMN levels varied across immune cell types, and reduced SMN levels were associated with altered immune cell composition. Immune alterations and decreased SMN levels were observed in both treated and untreated patients with SMA and may have contributed to dysfunctions of the immune system.

## Abstract 2

SMN expression varies across immune cell populations. In PBMCs of SMA patients, SMN is reduced and associated with persistent changes in immune cell composition.

## Introduction

The survival motor neuron (SMN) protein is a ubiquitously expressed protein with multiple functions in the nervous system and peripheral tissues. Complete loss of SMN is embryonically lethal, whereas partial loss, caused by deletions or mutations in the *SMN1* gene, impairs functional SMN expression and causes the neurodegenerative disease spinal muscular atrophy (SMA) (1, 2). The nearly identical *SMN2* gene cannot fully restore functional SMN levels (3, 4). *SMN2* differs from *SMN1* by mutations, including a critical C to T transition in exon 7, which causes SMN2 to produce only approximately 10%–20% full-length transcripts, while the remaining 80%–90% are spliced into a truncated, unstable isoform lacking exon 7 (*SMNΔ7*) (4, 5). Untreated, SMA is one of the leading genetic causes of infant mortality and is characterized by the degeneration of α motor neurons in the spinal cord, leading to progressive muscle weakness and atrophy (6, 7). Without therapeutic intervention, patients with the most severe form of SMA (formerly designated as type I) typically die within the first year of life (8). Currently available treatments increase SMN protein expression, substantially reduce disease burden, improve motor function, and augment survival (9–11). However, SMN-enhancing therapies do not cure SMA, and patients still present with a wide range of clinical symptoms, reflecting the broad systemic role of SMN across multiple organ systems, including the immune system (12–19). Immune responses require extensive cell proliferation and differentiation — processes that depend on large-scale remodeling of RNA metabolism (20, 21). Because SMN contributes to several aspects of RNA metabolism, including pre-mRNA splicing and the assembly of the spliceosomal small nuclear ribonucleoprotein particles (snRNPs), SMN deficiency may directly impair immune cell functions (22–27).

In WT mice, SMN levels are high during embryonic development, decline postnatally in most tissues, but increase in the spleen, and remain stable in the thymus through P19, suggesting a sustained role of SMN in the immune system (28). Consistent with this, SMA mouse models exhibit pronounced morphological and functional abnormalities in primary and secondary lymphoid organs including smaller thymus and spleen size, alterations in mucosa-associated lymphoid tissue and immune cell frequencies, abnormal microarchitecture of the pulps, cytokine dysregulation, impaired T cell and B cell development, and decreased blood flow (18, 28–31). Analysis of human PBMCs revealed temporal fluctuations in SMN levels within the CD14^+^ myeloid cell fraction in individuals who had recently experienced respiratory infections (32). In addition, SMN levels were shown to steadily decline with age, with a more pronounced reduction observed in patients with SMA compared with healthy individuals (32). Early studies conducted before the licensing of systemic therapy reported reduced SMN levels in B and T cells of patients with SMA (33). Furthermore, postmortem analyses showed that 56% (5 of 9) of patients with type I SMA presented with a reduced spleen size and signs of developmental defects in the splenic immune compartment (28, 30). Recent transcriptomic analyses of PBMCs from infants with SMA identified immune dysregulation as a central aspect of SMA, marked by distinct gene expression changes, and indicated a transition from metabolic regulation to immune activation with an inflection point occurring at around 6–7 months of age (34).

Currently approved therapies for SMA in adults include the intrathecally administered antisense oligonucleotide nusinersen and the orally available small-molecule and splicing modifier risdiplam (35). Consequently, nusinersen primarily exerts its therapeutic effect within the CNS, whereas risdiplam is systemically distributed and active in both the CNS and peripheral tissues (35, 36). Despite its broader effect range, the effect of risdiplam on immune cells has not been extensively studied. Intravenously administered adenovirus-associated (*AAV9*) gene replacement therapy with onasemnogene abeparvovec-xioi has been approved for children with type I SMA, biallelic mutations in the *SMN1* gene, and maximal 3 *SMN2* gene copies (37). Furthermore, in November 2025, the FDA approved onasemnogene abeparvovec-brve, an intrathecally administered therapy adapted from onasemnogene abeparvovec-xioi for the treatment of both pediatric and adult patients with SMA, but patient samples were not yet available at the time of this study (38). Collectively, the current evidence suggests that SMN contributes to immune cell biology not only during embryogenesis but also after birth. However, a thorough analysis of SMN abundance and distribution across immune subsets, during differentiation, and in response to treatment is still incomplete. In addition, the role of SMN in splicing regulation within immune cells remains underexplored. Addressing these gaps is essential for understanding peripheral manifestations of SMA and their potential effect on therapeutic efficacy.

The aim of this study was to characterize SMN expression across immune cell subsets and to examine how SMN levels change during immune cell differentiation. We studied immune cells of both mouse and human origin and compared SMN expression in nusinersen- and risdiplam-treated patients with SMA to evaluate potential immune-related effects of the therapies. Our findings reveal considerable variation in SMN levels across immune cell subsets and highlight the observation that SMN protein expression plays an ongoing role in maintaining immune homeostasis throughout life. Notably, compared with patients treated with nusinersen, patients treated with risdiplam exhibited an immune cell distribution more similar to that of healthy controls. However, both cohorts still showed altered PBMC frequencies and reduced SMN levels compared with healthy controls. Exploratory analysis of untreated patients with SMA confirmed that SMN deficiency and immune alterations were comparable to those observed in patients treated with risdiplam or nusinersen. Overall, these findings suggest that both therapies have only limited effects on the peripheral immune compartment, as immune cell imbalances persist in adult patients with SMA and may continue to affect immune function and inflammatory regulation.

## Results

### SMA mice from the Taiwanese mouse model show reduced splenic B cell numbers early after birth.

In humans, the *SMN* locus typically contains 1 copy of *SMN1* and a variable number of *SMN2* copies on each allele (ranging from 1 to ~8) (3, 39, 40). Development of SMA results from a homozygous deletion of, or mutations in, the *SMN1* gene (4). In contrast to humans, mice only have 1 *Smn* gene, and *Smn* KO is embryonically lethal (41, 42). Notably, *Smn*-deficient mice that carry 1 transgene allele with 2 human *SMN2* gene copies (S*mn^–/–^* *SMN2^tg/0^*) display a disease phenotype reminiscent of severe type I SMA. This model, known as the Taiwanese SMA mouse, produces litter that is 50% *Smn^–/–^* *SMN2^tg/0^* (SMA mice) and 50% *Smn^+/–^* *SMN^tg/0^* (littermate controls), the latter of which do not develop disease by P3 (Figure 1A) (41).

To analyze the immune cell composition of the premature spleen, we performed multicolor spectral flow cytometry on splenocytes isolated from P3 Taiwanese SMA mice. Briefly, spleens were extracted, mechanically dissociated into single-cell suspensions, and labeled with fluorescently conjugated antibodies directed against the lineage markers B220, CD3, CD4, CD8, CD11b, CD11c, F4/80, Ly6C, Ly6G, NKp46, CD86, and CD206 (Figure 1A, steps 2–5, and Supplemental Figure 1A; supplemental material available online with this article; https://doi.org/10.1172/JCI203540DS1). Dimensionality reduction (uniform manifold approximation and projection [UMAP]) analysis revealed that P3 splenocytes were primarily composed of B cells, monocytes, and neutrophils (Figure 1, B and C). Although the same major immune cell clusters were identified in SMA mice and littermate controls (Figure 1D), SMA mice exhibited markedly reduced B cells and increased neutrophils (Figure 1E). Relative quantification of splenic immune cell subsets showed significantly decreased B cells (10.7% vs. 29.2% in controls) and DCs (0.3% vs. 0.7% in controls), as well as increased neutrophils (23.5% vs. 10.2% in controls), macrophages (15.3% vs. 9.8% in controls), and CD8^+^ T cells (0.8% vs. 0.4% in controls) (Figure 1F). Analysis of absolute cell numbers confirmed the significant decrease in B cells, DCs, and CD4^+^ T cells, and the increase in neutrophils and macrophages, whereas absolute CD8^+^ T cell counts did not differ between SMA mice and control littermates (Figure 1G). Together, these results indicate a loss of B cells and an increase in neutrophils and macrophages in spleens of P3 SMA mice.

### Absolute SMN protein levels vary across splenic immune cell subsets from mice at P3, with B cells showing the highest expression.

To assess SMN protein levels in different immune cell subsets of P3 spleens, we measured intracellular SMN expression by spectral flow cytometry. Quantification of the geometric MFI (gMFI) in control mice showed SMN expression across all analyzed cell subsets, with particularly high SMN levels in the B cell cluster (Figure 2A). SMN expression profiles of individual animals indicated 2 distinct cell populations characterized by low and high protein levels (Figure 2B, left). Analysis of specific immune cell subsets further demonstrated that B cells and macrophages expressed the highest SMN levels and CD4^+^ T cells and neutrophils the lowest SMN levels (Figure 2B, right).

In SMA mice, SMN expression mapped to a cluster similar to that observed in control littermates (Figure 2C). However, the independently normalized histogram revealed a relative decrease in cells with high SMN levels in SMA animals when compared with controls (Figure 2D, left). This observation is consistent with the reduced abundance of B cells (Figure 1, F and G), which likely contributed to the diminished SMN-high signal. Furthermore, as observed in control mice, B cells and macrophages showed the highest SMN expression in SMA animals (Figure 2D, right), whereas quantification of SMN levels among all live cells indicated that F4/80^+^ macrophages and monocytes from SMA mice expressed significantly lower SMN levels than did the corresponding cell subsets in littermate controls (Figure 2E). In contrast, the immunosuppressive CD206^+^ F4/80^+^ macrophage subset showed even higher SMN levels in SMA mice than in littermate controls (Figure 2E). To further determine whether SMN levels are linked to immune cell differentiation, we analyzed additional markers including CD80, Ly6C, and MHC-I/-II, which are associated with myeloid cell stimulation and differentiation (Supplemental Figure 1B). Among these markers, only MHC-I showed a moderate positive correlation with SMN expression in control mice. However, further mechanistic studies are required to determine whether this association has functional relevance.

The high SMN expression in B cells and macrophages, together with the relative loss of the SMN-high peak in SMA mice, prompted us to perform a more detailed analysis of immune cell subsets within the SMN-low and SMN-high populations (Supplemental Figure 2A). Of note, the majority of cells lost in P3 spleens of SMA mice belonged to the SMN high population (Supplemental Figure 2B), which included both B220^+^ B cells and F4/80^+^ macrophages (Supplemental Figure 2, C and D). As shown above, in SMA animals, B cells were significantly decreased and macrophages were increased, regarding both their relative proportion and absolute cell counts, suggesting that the loss of the SMN-high population resulted primarily from the reduction in B cells rather than macrophages (Figure 1, F and G). In summary, B cells and macrophages expressed the highest SMN levels in the spleens of P3 control animals. In SMA mice, SMN levels were significantly reduced in F4/80^+^ macrophages and monocytes and increased in CD206-expressing macrophages. Although SMN levels in B cells of SMA mice were largely maintained, the overall number of B cells was reduced.

### B cell numbers are increased in PBMCs of adult patients with SMA.

To determine potential alterations in the immune cell composition of patients with SMA, we performed spectral flow cytometry of PBMCs from 23 adult patients with SMA and 16 healthy controls (Figure 3A). Immune cell populations were identified by the expression of established cell-surface markers (Supplemental Figure 3A). UMAP projections of PBMCs revealed distinct clusters corresponding to B cells, T cells, NK cells, monocytes, DCs, and basophils (Figure 3B). Interestingly, patients with SMA displayed a markedly altered PBMC subset distribution when compared with healthy controls (Figure 3C). Specifically, quantification of CD45^+^ cells showed that B cells and CD4^+^ T cells were significantly increased, whereas CD8^+^ T cells and NK cells were significantly reduced in patients with SMA (Figure 3D). In addition, monocytes were moderately increased, although the values varied among individual patients (Supplemental Figure 4, A and B). Given the alterations in T cell subsets of patients with SMA, we next examined the activation status of professional antigen-presenting cells by measuring the expression of MHC and costimulatory molecules. On cDC1, MHC-I expression (gMFI of HLA-A, -B, and -C [HLA-ABC]) was slightly elevated, whereas MHC-II expression (gMFI of HLA-DR) was reduced on monocytes and cDC2 (Figure 3, E and F). The costimulatory molecule CD86 was not significantly changed (Figure 3G), and CD40 expression levels were comparable between patients with SMA and controls (Figure 3H). In summary, PBMCs from patients with SMA were characterized by increased B cell and CD4^+^ T cell frequencies along with decreased CD8^+^ T cell and NK cell frequencies.

### SMN protein levels are reduced in DCs from adult patients with SMA.

Overall, SMN expression was globally reduced across all analyzed immune cell subsets in patients with SMA relative to healthy controls (Figure 4A). In PBMCs of healthy controls, we detected particularly high SMN levels in monocytes and DC clusters (Figure 4B), which was in contrast to the murine P3 spleen, in which B cells displayed the highest SMN expression (Figure 2A). Additionally, while P3 mouse splenocytes showed 2 distinct SMN expression peaks (Figure 2B, left), human PBMCs displayed a single, relatively broad peak (Figure 4C, left). Analysis of single immune cell subsets confirmed that monocytes and DCs showed the highest SMN expression in healthy controls (Figure 4C, right). Despite overall reduced SMN levels in patients with SMA (Figure 4A), the distribution of SMN across immune cell clusters matched that of healthy controls (Figure 4D). Importantly, SMN expression levels were reduced in all but 1 patient with SMA when compared with healthy controls (Figure 4E, left, Supplemental Figure 5). As shown for a representative risdiplam-treated patient, DCs exhibited the strongest reduction of SMN levels compared with controls, whereas the other immune cell subsets were less affected (Figure 4E, right). Additionally, higher SMN expression levels were associated with increased HLA-ABC and decreased CD80 expression, whereas no significant correlation was observed for HLA-DR or CD40 (Supplemental Figure 3, B and C). Taken together, in PBMCs of healthy controls, DCs and monocytes expressed the highest SMN levels. In contrast, in patients with SMA, SMN expression was reduced across all immune cells, and particularly in DCs; however, DCs and monocytes still retained the highest SMN levels relative to other immune cell subsets.

### Risdiplam- and nusinersen-treated patients exhibit comparable immune profiles, with persistent divergence from healthy controls.

To assess how SMN-targeted therapies influence the peripheral immune cell composition and SMN expression, we analyzed PBMCs from patients with SMA treated with either nusinersen or risdiplam. Nusinersen is administered intrathecally and does not cross the blood-brain barrier (BBB), which results in CNS-restricted activity. Risdiplam, on the other hand, is administered orally, passes the BBB, and exerts systemic effects (35, 43). The studied SMA cohort included patients with type II and III SMA (Figure 5A and Supplemental Table 1). Importantly, patients with SMA and healthy controls were similar in age (Figure 5B) and showed a comparable sex distribution (Figure 5C). Furthermore, clinical motor scores aligned with the ambulatory status of the patients (Figure 5D). Overall, the cohort comprised 15 nusinersen- and risdiplam-treated patients (Figure 5E). PBMCs of all patients showed reduced SMN expression relative to that in healthy control PBMCs; however, the decrease in SMN expression reached statistical significance only in the nusinersen-treated group (Figure 5E). Comparison of *SMN2* copy numbers with SMN levels in individual patients revealed a positive correlation (Figure 5F), which is in accordance with the established contribution of the *SMN2* locus to full-length stable SMN expression in patients with deleted or mutated *SMN1*. To determine whether treatment with risdiplam, which enhances SMN expression from the *SMN2* gene systemically, influences this relationship, we analyzed each treatment group separately. However, within the groups, we found that *SMN2* copy numbers did not correlate with SMN levels, probably because of the limited number of patients analyzed (statistics not shown). As SMN levels in PBMCs of healthy individuals have previously been reported to decrease with age, i.e., between infancy and adulthood (32, 44, 45), we next analyzed the relationship between SMN expression and age in adults. Surprisingly, SMN levels in healthy controls showed a significant positive correlation with increasing age (Figure 5G). To ensure that age differences did not confound comparisons between treatment groups, we calculated the average age of patients, which was 42.6 years (25–70 years) for the nusinersen-treated group and 36.9 years (18–57 years) for the risdiplam-treated group. The healthy control group had an average age of 35.9 years (19–64 years). These findings indicate that age differences are unlikely to account for the reduced SMN levels observed in patients with SMA or variations between treatment groups. Since DCs showed the highest SMN expression levels in PBMCs, we additionally correlated their frequency with patient age. In healthy individuals, DC frequency significantly increased with age, potentially contributing to the age-associated increase in SMN levels observed in CD45^+^ cells (Supplemental Figure 3D).

Evaluation of the PBMC subset composition demonstrated that nusinersen-treated patients showed a significant increase in CD4^+^ T cells and a decrease in CD8^+^ T cells and NK cells compared with healthy controls (Figure 5H). SMN levels were significantly decreased in all immune cell subsets, with the exception of CD4^+^ T cells (Figure 5I). In contrast, PBMCs from risdiplam-treated patients showed no significant deviations from controls, even though subset frequencies and SMN levels did not fully reach control levels. Finally, we analyzed the correlation of clinical motor scores (Hammersmith Functional Motor Scale [HFSME] and Revised Upper Limb Module [RULM]) with SMN levels in patients, but high inter-individual variations prevented conclusions (Supplemental Figure 3, E and F). Thus, our data suggest that risdiplam*-*treated patients had immune cell frequencies that were more similar to those of healthy controls than nusinersen-treated patients, indicating a potentially greater systemic effect. However, conclusions regarding treatment-specific effects are limited by the lack of longitudinal samples and untreated controls.

### Untreated and treated patients with SMA display comparable SMN deficiency and immune cell alterations.

To distinguish disease-associated from treatment-associated effects on PBMC composition and SMN expression, we performed an exploratory analysis including untreated patients with SMA (Figure 6A). As disease-modifying treatments are widely available in Europe, untreated samples from adult patients with SMA are rare and were therefore not included in the primary cohort. Samples were matched when possible (Supplemental Table 2). In addition, samples from treated patients with SMA included in the primary cohort were reanalyzed together with the untreated samples and yielded comparable results, supporting the reproducibility of the findings (analysis not shown).

SMN expression levels in total CD45^+^ cells were similarly reduced in treated and untreated patients with SMA, although the differences compared with healthy controls did not reach significance. Slight deviations from results shown in Figure 3D and Figure 4A are probably due to the smaller sample size and the inclusion of a single SMA type I patient sample (red asterisk) (Figure 6B). Analysis of individual immune subsets showed significantly reduced SMN levels in untreated patients with SMA and in most subsets from treated patients with SMA compared with healthy controls (Figure 6C). Therefore, we conclude that both SMN-enhancing therapies appeared unable to sustain SMN levels across immune cell populations comparable to the levels observed in healthy controls. Nevertheless, histogram analysis confirmed that monocytes and DCs consistently exhibited the highest SMN expression across all analyzed sample groups (Figure 6D). As shown previously, B cells were significantly increased and CD8^+^ T cells were decreased within the CD45^+^ compartment of treated patients with SMA (Figure 3D). Importantly, untreated and treated patients with SMA showed similar immune cell alterations relative to healthy controls (Figure 6E). In conclusion, this exploratory analysis provides additional evidence that reduced SMN levels and altered immune cell distributions persisted in PBMCs from adult patients with SMA despite treatment.

### Aberrantly spliced SMNΔ7 mRNA levels decrease and SMN accumulates in spliceosome biogenesis–associated Cajal bodies during THP-1 cell differentiation.

Since monocytes and monocyte-derived myeloid cell subsets expressed the highest SMN levels among human PBMCs (Figure 4B), we performed exploratory experiments to determine the role of SMN in human monocytic THP-1 cells (Figure 7A). PMA-induced differentiation of THP-1 cells (PMA–THP-1) was accompanied by a shift from suspension to adherent growth, along with pronounced morphological changes (Figure 7A).

To examine the effect of differentiation on *SMN* mRNA splicing, we analyzed both the full-length properly spliced SMN mRNA isoforms of *SMN1* and *SMN2*, which encode the functional SMN protein, and the alternatively spliced isoform lacking exon 7 (*SMNΔ7*), which accounts for approximately 80% of *SMN2* transcripts and produces a shortened, less stable protein. Consequently, lower levels of full-length *SMN* could indicate a reduced abundance of functional protein, thereby limiting snRNP assembly activity (3, 46). Surprisingly, full-length SMN mRNA levels remained stable during THP-1 differentiation and cytokine stimulation, but SMNΔ7 levels decreased, as assessed by quantitative agarose gel electrophoresis (*n* = 1) (Figure 7B). We next analyzed SMN protein expression during differentiation of THP-1 and NSC34 cells, the latter of which is an immortalized motor neuron–like cell line commonly used to study SMN biology (47, 48). Despite high basal SMN expression in NSC34 cells, differentiation of both cell types did not affect SMN protein levels, as shown by Western blot analysis (Figure 7, C and D). The SMN protein localizes to multiple cellular compartments, including distinct subnuclear structures known as coilin^+^ Cajal bodies and coilin^–^ gems, both of which contain components of the Gemin complex (49, 50). Cajal bodies additionally contain snRNPs and are implicated in tri-snRNP biogenesis (24, 51). Confocal imaging of SMN subcellular localization revealed its presence in Cajal bodies and gems across all analyzed cell types, including untreated THP-1 cells, PMA–THP-1 cells, and IFN-γ–/TNF-α–stimulated PMA–THP-1 cells (Figure 7, E and F). Notably, PMA-induced differentiation of THP-1 cells was associated with a significant increase in the maximum intensity projection of the SMN signal (not quantified) and a higher number of SMN^+^ Cajal bodies, indicating enhanced SMN involvement in tri-snRNP assembly during THP-1 cell differentiation. In summary, differentiation does not alter total SMN protein levels in THP-1 or NSC34 cells but is associated with reduced levels of the aberrantly spliced SMNΔ7 mRNA, which suggests a shift in the regulation of *SMN* gene expression or SMNΔ7 mRNA decay. Moreover, PMA-induced differentiation results in an increased number of SMN^+^ Cajal bodies indicating enhanced recruitment of SMN to snRNP maturation sites. These exploratory experiments give a first impression on the importance of SMN during differentiation, although additional studies are required to define its precise function in primary immune cells.

## Discussion

SMA is a rare monogenic disease caused by reduced levels or functional impairment of the SMN protein (3, 4). Although SMA is primarily linked to motor neuron degeneration, increasing evidence indicates that SMN also plays important roles in peripheral tissues, such as the immune system (18, 28–30). In this study we analyzed SMN expression across immune cell subsets and following nusinersen or risdiplam treatment of adult patients with SMA. Given the high demands for SMN during murine embryogenesis, we first assessed SMN expression in immune cells shortly after birth using the murine Taiwanese SMA model (1, 41). In this model, spleen size and total splenic cell numbers are normal at birth (P0) but decrease dramatically when mice reach symptomatic stages (P5–P8) (30). To study early immune defects, we performed immunophenotyping of the developing spleen at P3 using spectral flow cytometry (52). Our data reveal that SMN expression was highly cell-type specific within the spleen and that SMA disrupted immune cell homeostasis. Neonatal SMA mice showed pronounced alterations in the splenic immune cell composition, including a marked reduction in B cells and an increase in neutrophils. B cells and macrophages expressed the highest SMN levels among all cell subsets, however, the loss of the SMN-high population was primarily driven by the profound reduction of B cells rather than macrophages. Overall, these findings extend earlier observations of lymphoid organ defects in SMA mouse models and indicate that insufficient SMN affects the development and maintenance particularly of B cells during early life. Our results differ from observations made in the *SMNΔ7* mouse model, a model of severe SMA in which affected mice on the *Smn^–/–^* background homozygously carry a transgene containing the human *SMNΔ7* and *SMN2* genes (Smn^–/–^ *SMNΔ7:SMN2^tg/tg^*) (42). This additional *SMNΔ7* transgene distinguishes *SMNΔ7* mice from the Taiwanese SMA mice analyzed in our study. In the *SMNΔ7* model, a relative increase in B cell numbers has been reported, likely due to a loss of red pulp tissue-resident cell populations at the symptomatic time point P12 (29). Another recently published study reported an abnormal distribution of B cells and T cells and an expansion of resident macrophages at P14 in *SMNΔ7* mice (31). The authors attribute these changes to developmental defects in the bone marrow and impaired immune cell maturation in the thymus rather than increased apoptosis, highlighting immune dysregulation as a key feature of SMA. Notably, these immune cell alterations were largely prevented by early treatment with a nusinersen-like antisense oligonucleotide. In contrast, histological analyses of the less severely affected SMA *Smn^2B/–^* mice, in which 1 allele of murine *Smn* is knocked out and the other allele carries a knockin mutation of a splice enhancer in exon 7, showed a diffuse localization of B cells and macrophages in the white pulp of the spleen at the symptomatic day P19 (28, 53). Importantly, this anatomical defect was not observed in the same study in Taiwanese SMA mice at P2 and P5 (28). These distinct patterns in B cell frequencies and localization across SMA models may result from differences in disease severity or progression at the time of analysis. In our study, both absolute and relative quantification consistently demonstrated a significant reduction in B cells. Therefore, our findings, together with previous studies, support an association between reduced SMN levels and immune imbalance, although further mechanistic studies are needed to draw a conclusion on direct causality.

In contrast to the neonatal murine spleen, adult patients with SMA displayed increased B cell numbers in PBMCs. However, direct comparisons between species were limited by differences in tissue type, disease severity and stage, species-specific characteristics, and genetic background. Moreover, increased B cell frequencies within the normal PBMC range do not necessarily imply preserved B cell function in patients with SMA, as altered activation states or functional impairment may still be present. These observations align with the concept of SMA as a multisystem disease, in which peripheral organ dysfunction may contribute to altered immune homeostasis (12, 54–56). Hepatocytes, for instance, exhibit intrinsic SMN-dependent defects associated with hepatic steatosis in patients with SMA, potentially affecting immune regulation through interactions within the liver-immune axis (57).

Detailed immune profiling in our patient cohort further revealed increased frequencies of CD4^+^ T cells and decreased CD8^+^ T and NK cells, indicating broader changes in the lymphoid cell compartment. In contrast, single-cell transcriptomics analysis of infants with SMA (<25 months of age) identified reduced CD4^+^ T cells alongside increased CD8^+^ T cells and NK cells. Pathway analysis further suggested a pre-exhaustion state in CD4^+^ T cells and dysfunctional CD8^+^ T cell responses associated with the MHC-I pathway in symptomatic infants (34). In our PBMC dataset, monocytes and DCs showed the highest SMN expression across controls and patients with SMA, potentially reflecting elevated transcriptional and splicing demands of professional antigen-presenting cells. Similarly, previous studies have shown high SMN expression in CD14^+^ monocytes from patients with recurrent respiratory infections, suggesting higher SMN demands in monocytes during immune activation (32). A study that investigated SMN as a biomarker for treatment response demonstrated that total PBMCs from patients with SMA show lower SMN levels than in PBMCs from controls, as determined by ELISA (45). In addition, image-based assays revealed lower nuclear SMN fluorescence intensity in CD19^+^, CD3^+^, and CD33^+^ cells from both infants and adult (*n* = 3) patients (33). In accordance with these results, we found that patients with SMA displayed a global reduction of SMN across all PBMC subsets (B cells, T cells, NK cells, monocytes, DCs and basophils). Although DCs exhibited the most pronounced decrease, they still retained higher SMN levels compared with other immune cell subsets. Importantly, patients with SMA exhibited increased HLA-ABC expression on conventional dendritic cells type 1 (cDC1s) (Figure 3E) despite reduced SMN levels. In contrast, analysis of total live or CD45^+^ cells revealed a positive correlation between SMN and HLA-ABC/MHC levels in both patients with SMA and control mice. These seemingly divergent findings likely reflect differences in the analyzed immune cell populations, as well as developmental, tissue-specific, and species-dependent factors that may differentially influence antigen presentation pathways. Notably, the apparent difference in HLA-ABC expression was not retained upon treatment stratification, highlighting the limited robustness of this observation and the constraints imposed by small sample size. Further mechanistic studies will be required to determine whether reduced SMN directly affects antigen presentation and costimulation.

Currently, adult patients with SMA can be treated with 2 SMN-enhancing therapies, nusinersen and risdiplam, which increase SMN protein levels by modulating *SMN2* splicing. While both treatments improve and/or stabilize motor function, only risdiplam efficiently reaches peripheral tissues (58). Despite this pharmacological difference, both treatment groups exhibited lower SMN levels compared with healthy controls; however, the decrease was not statistically significant in risdiplam-treated patients. Nevertheless, immune cell subset distribution of risdiplam-treated patients more closely resembled that of healthy controls, whereas nusinersen-treated patients, whose treatment was restricted to the CNS, displayed stronger alterations in T cell and NK cell frequencies (35, 36). Although limited by the small number of untreated adult patients with SMA, exploratory analyses allowed the direct assessment of baseline immune abnormalities. Comparison of untreated and treated (nusinersen or risdiplam) patients with SMA revealed no major differences in immune cell composition or SMN expression, suggesting that neither therapy exerted substantial immunomodulatory effects in this cohort. Given the small cohort size, we could not reliably assess treatment-specific pharmacological effects. Interestingly, changes in immune cell composition may be accompanied by functional alterations, as T cells and NK cells in the cerebrospinal fluid (CSF) of patients with SMA were reported to exhibit cytotoxic activity, which was reduced following nusinersen treatment (59). Furthermore, bulk RNA-seq analyses following AAV9-based therapy revealed increased immune activation with elevated TNF-α/NF-κB signaling, enhanced neutrophil degranulation, and increased abundance of M2 macrophages between 0.9 and 8 months after treatment (34). Together, these findings highlight the importance of considering peripheral immune alterations in SMA and suggest that systemic restoration of SMN may be required to effectively target both motor dysfunction and immune-related phenotypes. This concept may be particularly relevant as AAV-based therapies become available for adult patients (37, 60). Additional clinical confounders — including disease duration, functional status, treatment duration, and recent infections or vaccinations — could not be fully accounted for and may have influenced the observed immune profiles. Autoantibody measurements performed in a subset of patients with SMA did not reveal specific abnormalities (Supplemental Table 2). SMN expression in patients is influenced by copy number variations of *SMN2*, yet protein levels do not always correlate strictly with the *SMN2* copy number, further highlighting structural variations in the SMN locus (46, 61). In our cohort, we observed a significant positive correlation of SMN protein levels and *SMN2* copy numbers in PBMCs of patients with SMA, although the distinction by treatment group was not significant, probably due to the limited number of patients studied (nusinersen: mean copy number: 3.3, *n* = 15; risdiplam: mean copy number: 3.8, *n* = 5, data for 3 patients missing, 1 individual with 6 copies). Among the analyzed patients with SMA, the highest SMN gMFI values were observed in a risdiplam*-*treated individual who carried 6 *SMN2* gene copies (Supplemental Table 1 and Figure 5). As this patient represents a rare but clinically relevant subgroup, the patient was retained in the analysis rather than excluded as an outlier. Nevertheless, exclusion of this sample resulted in significantly lower SMN levels in total CD45^+^ cells compared with controls (Figure 5E) and rendered most immune cell subset comparisons statistically significant (*P* < 0.05), except for monocytes and CD4^+^ T cells. Thus, these analyses indicate that reduced SMN levels are also present in PBMCs of most risdiplam*-*treated patients. Together, these findings emphasize the persistence of immune alterations in SMA despite current therapies and highlight the need for further investigation of immune dysfunction and complementary treatment strategies.

In PBMCs from healthy adults, we found increasing SMN levels with age, raising the possibility that chronic SMN deficiency may contribute to immune vulnerability in older individuals. In contrast, previous ELISA-based studies reported highly variable SMN protein levels and an age-dependent decline of SMN from infancy and during adulthood in patients with SMA, healthy controls, or *SMN1* mutation carriers (32, 45). These discrepant findings may reflect differences in clinical covariates, tissue source, immune cell composition, and cellular resolution, underscoring the need for larger, stratified cohorts to better assess age-dependent changes in SMN expression. In our dataset, DCs exhibited high SMN expression and increased with age in PBMCs of healthy controls, thus possibly contributing to the observed correlation between SMN levels and age (Supplemental Figure 3D). Evidence from mouse models further support a continued requirement for SMN in the immune system. While SMN expression decreases in most peripheral tissues early after birth, it remains stable or increases in secondary lymphoid organs until P19 (28). In the SMA *Smn^2B/-^* and the *SMNΔ7* mouse model, introduction of a single human *SMN2* transgene or treatment with an antisense oligonucleotide, respectively, was sufficient to normalize secondary lymphoid organ development (28, 31). Together, these findings suggest that adequate SMN levels after birth and throughout adulthood are critical for maintaining immune system integrity. However, the precise molecular functions of SMN in immune cell biology in vivo remain an objective for future research.

To further explore the mechanistic role of SMN in myeloid cells, we used the human monocytic THP-1 cell line as a model system. Given the high expression of SMN observed in monocytes and monocyte-derived cells as well as SMN’s essential function in snRNP assembly, we investigated how SMN is regulated during PMA-induced THP-1 differentiation, a process that is associated with high splicing activity (25, 27, 51). Although total SMN protein levels remained stable, *SMNΔ7* transcript levels decreased during THP-1 cell differentiation. This, along with an increased number of SMN^+^ snRNP maturation–associated Cajal bodies, suggests enhanced splicing activity, which is probably important for immune cell maturation. However, the precise role of SMN in immune cell differentiation throughout life remains unclear. The observed changes in SMN localization in THP-1 cells suggest a potential involvement of SMN in these processes, although the functional consequences of SMN deficiency require further investigation.

In this study, we analyzed SMN expression across diverse immune cell subsets of mouse and human origin to assess cell-type–specific differences in SMN levels, their changes during immune cell differentiation, and the effect of altered SMN expression and/or function on the peripheral immune cell composition. Our findings reveal substantial heterogeneity in SMN expression across immune cell populations and highlight that adequate SMN protein levels are required throughout life to maintain immune homeostasis. In addition, our data raise the possibility that cells with inherently high SMN levels, such as murine B cells and human DCs, are particularly vulnerable to SMN reduction. Therefore, we hypothesize that individual immune cell types differ in their SMN requirements, and those with higher baseline demands may reach critically low levels more readily under SMA conditions. Importantly, this study is limited in its ability to draw conclusions regarding developmental aspects of immune dysregulation in SMA due to a lack of samples from human neonates, severely affected patient cohorts, and sufficient amount of blood from P3 SMA mice. To gain preliminary insight into immune alterations early in disease, PBMCs from a single treatment-naive infant with SMA were analyzed by flow cytometry. Compared with adult patients with SMA, the infant sample showed a lower frequency of monocytes. Notably, monocyte SMN levels were also reduced relative to both adult healthy controls and treated adult patients with SMA (Supplemental Figure 6). Despite these limitations, our results provide evidence that systemic SMN restoration may offer broader therapeutic benefits for patients beyond motor function recovery, particularly by helping to preserve immune homeostasis. Altogether, this study reinforces the view of SMN as a fundamental multifunctional protein and underscores the need for treatment strategies that address its systemic roles to fully meet the complex clinical needs of individuals with SMA.

## Methods

### Sex as a biological variable

This study includes both human and animal data. Neonatal mice were examined on P3. At this developmental stage, sex cannot be reliably determined by morphological assessment; therefore, sex was not assigned. Human samples were obtained from both male and female donors, and SMA and control cohorts were matched for sex distribution. Although donor sex was recorded and cohorts were balanced for sex, sex-stratified analyses were not performed because of the limited cohort size.

### Cell culture

NSC34 cells (from Gabriella Viero, Institute of Biophysics, CNR Unit at Trento, Trento, Italy), a motoneuron-like cell line derived from murine neuroblastoma and spinal cord cells, were cultivated in DMEM with GlutaMAX-I with 5% FCS (Thermo Fisher Scientific, 10500-064) and 1% penicillin-streptomycin (100 U/mL, Gibco, Thermo Fisher Scientific, 15140122) at 37°C and 5% CO_2_. Cells were seeded at 3 × 10^5^ cells/well in 6-well plates and cultured in starvation medium with 1% FCS for 48 hours prior to analysis. THP-1 cells (TIB-202, American Type Culture Collection [ATCC]), a leukemia-derived human monocytic–like cell line, were cultivated in RPMI 1640 Medium (Gibco, Thermo Fisher Scientific, 31870025) containing 10% FCS (Thermo Fisher Scientific, 10500-064), 1% GlutaMAX Supplement (Gibco, Thermo Fisher Scientific, 35050061), and 1% penicillin-streptomycin (100 U/mL, Gibco, Thermo Fisher Scientific, 15140122) at 37°C and 5% CO_2_. For differentiation into adherent macrophages, THP-1 monocytic cells were stimulated with 20 ng/mL phorbol 12-myristate 13-acetate (PMA) in DMSO for 24 hours. Then, THP-1–derived cells (PMA–THP-1) were rested for 72 hours in PMA-free medium or incubated with 20 ng/mL IFN-γ and 100 ng/mL TNF-α for 48 hours to induce a proinflammatory state. As control, THP-1 cells were treated with equal (PMA) volumes of DMSO (1 μL/mL).

### Western blotting

NSC34 cells were seeded and cultivated as described above. THP-1 cells for differentiation were seeded with 2 × 10^5^ cells/6-well plate and undifferentiated controls with 0.5 × 10^5^/6-well plate in 2.5 mL medium. After culturing as described above, cells were washed 3 times with PBS at room temperature (RT) and lysed with 200 μL RIPA buffer/well including 1× cOmplete Protease Inhibitor Cocktail (Roche, 04693132001) at 4°C. Cells were allowed to swell on ice for 15 minutes, harvested by scraping, and passed through a 27 gauge needle using a syringe. Cell debris was pelleted by centrifugation at 21,000*g* for 10 minutes ,and 4°C and protein concentrations were determined from supernatant with the Pierce BCA Protein Assay Kit (Thermo Fisher Scientific, 23227). Läemmli buffer (5×) was added to 40 μg sample, incubated at 95°C for 5 minutes, cooled down, and stored at –20°C. Samples were loaded on 10% division gel with 5% stacking gel and run at 160 V. Proteins were wet-transferred to a nitrocellulose membrane (Amersham VWR, REF 10600003, 0.45 μm) for 1 hour at 120 V in a cooled container. Total protein staining was performed by Ponceau S solution for 5 minutes at RT. The background was removed with distilled H_2_O. After total protein detection, staining was removed by TBS-Tween 20 (TBS-T). Primary antibodies (anti-SMN, BD Transduction Laboratories, 601647, 1:3,000 in 5% milk in TBS-T) were incubated overnight at 4°C by rotation followed by the secondary antibody [ECL Anti–mouse IgG HRP linked F(ab)2 fragment (Cytiva, REF NA9310), 1:5,000 in 5% milk in TBS-T] for 1 hour at RT. Membranes were developed by Immobilon Western Blot Reagent (MilliporeSigma, WBKLS0500). Quantification was performed with Labimage1D software (Kapelan, version 7.1.1), whereby signal intensities were normalized to the geometric mean of each membrane and to total protein.

### Quantitative real-time PCR

PMA–THP-1 and THP-1 cells were seeded at 2 × 10^5^ cells/well and 0.5 × 10^5^ cells/well in 6-well plates. After culturing as described above, cells were washed with PBS, lysed in RLT buffer, and genomic DNA was fragmented by passing the lysate 10 times through a 27 gauge needle. RNA was isolated using the Qiagen RNA Mini kit and cDNA was transcribed using the iScript cDNA synthesis Kit (Bio-Rad, 1708890) according to the manufacturer’s instructions. Primers were designed to span exons 6–8 (hSMN_Ex6_F: 5′-GCTGATGCTTTGGGAAGTATGTTA-3′; 5′- ATTCCAGATCTGTCTGATCG-3′). Both primers were validated for specificity by Primer-BLAST (NCBI, version 2.5.0; https://blast.ncbi.nlm.nih.gov/Blast.cgi), melt curve analysis, and 3% agarose gel electrophoresis to confirm the correct product size. Quantitative real-time PCR (qRT-PCR) was performed with 50 ng cDNA/well, 1.75 μM per primer, 5 μL SYBR Green Mastermix, 0.1 μL CXR Reference Dye, and 0.4 μL RNAse-free H_2_O (Promega kit, A6020). StepOnePlus thermocycler was set to 10 minutes at 95°C, followed by 40 cycles of 15 seconds at 95°C and 1 minute at 60°C. Melt curve analysis was performed at the end of the run with 15 seconds at 95°C, 1 minute at 60°C, and 15 seconds at 95°C with a +0.3°C temperature ramp. Transcript sizes of different splicing isoforms were analyzed by subsequent agarose gel electrophoresis in a 3% gel.

#### Immunocytochemistry.

PMA–THP-1 cells and stimulated cells were seeded at 5 × 10^4^ cells/well on a glass coverslip, precoated for 1 hour at 37°C with PLL, and cultured as described above. Unstimulated THP-1 cells from the same passage were seeded as controls at 2 × 10^5^ cells/well in 500 μL PBS. Cells were allowed to settle for 30 minutes at RT, fixed with 4% PFA for 10 minutes at RT, washed 3 times with PBS, and stored at 4°C until staining. Differentiated and stimulated cells were fixed with 4% PFA for 10 minutes at RT and washed 3 times with PBS for 5 minutes. Staining was performed for each experiment set (cells with the same passage) separately. Cells were permeabilized with 1% Tergitol/1% (w/v) BSA/PBS for 15 minutes at RT, washed 3 times with 500 μL PBS for 5 minutes, and blocked with 5% NGS (v/v)/0.3% Tergitol in PBS for 60 minutes at RT. Primary antibodies anti-SMN (1:1,000, BD Transduction Laboratories, 610646) and anti-Coilin (1:1,000, Abcam, ab210785, 1C3) were diluted in 1% NGS (v/v)/0.3% Tergitol in PBS and incubated overnight at 4°C. On the next day, cells were washed 3 times with PBS for 5 minutes and incubated with secondary antibodies anti–mouse Alexa Fluor 555 (1:500, Thermo Fisher Scientific, A-21422) and anti–rabbit Alexa Fluor 488 (1:500, Thermo Fisher Scientific, A-11008) diluted in 1% NGS (v/v)/0.3% Tergitol in PBS for 1 hour at RT in the dark. Finally, cells were washed 3 times with PBS for 5 minutes, and nuclei were counterstained with DAPI (1:4,000) in PBS for 5 minutes at RT, washed again once with distilled water for 5 minutes, mounted with ProlongDiamond, and dried overnight at 4°C. All Images were acquired on a Zeiss LSM 780 microscope with ZEISS ZEN software (2.3 SP1). Slides were blinded, and single cells were imaged in predefined regions of interest. The maximum intensity projections were analyzed in a blinded manner and manually counted for SMN^+^, Coilin^+^, and double-positive bodies localizing in the nucleus (DAPI^+^).

### Taiwanese SMA model

Mice were housed in the central animal facility of the Hannover Medical School on a 12-hour light/12-hour dark cycle with ad libitum access to food and water. Taiwanese SMA mice (*FVB.Cg-Tg(SMN2)2Hung SMN1tm1Hung/J*, The Jackson Laboratory, stock no. 005058) were bred as described previously, resulting in 1:1 SMA and heterozygous control littermates (56). SMA mice have a homozygous KO allele of the murine *Smn* gene and carry 1 transgenic allele with 2 human *SMN2* copies. Heterozygous controls are heterozygous for the murine *Smn* and have 1 transgenic allele with 2 human *SMN2* copies. Mice were analyzed on P3, the onset of motoric symptoms and weight loss. Mice were decapitated according to the German animal welfare law, and spleens were dissected and stored in PBS on ice until flow cytometric analysis. Genotyping was done from tail snips according to the high-resolution PCR protocol from The Jackson Laboratory (https://www.jax.org/strain/005058, Primer 441-443).

### Study cohort and sample collection

Blood samples from patients with SMA and age-matched healthy controls were collected at the Department of Neurology and the Department of Rheumatology and Immunology at Hannover Medical School and at the TWINCORE – Centre for Experimental and Clinical Infection Research, following institutional ethics guidelines. Blood samples from untreated patients with SMA were collected at the Clinic for Neurology of University Hospital Essen (AöR). One blood sample from an untreated infant with SMA was collected at the Clinic for Pediatric Kidney, Liver, Metabolic, and Neurological Diseases at Hannover Medical School (32). Blood was drawn into EDTA tubes, stored under constant temperature (RT), and processed within 2 hours of collection. SMN levels are reported to be stable by this method as shown by ELISA (32). Clinical and demographic characteristics of healthy controls and patients with SMA are provided in Figure 5, A–D, Figure 6, and Supplemental Tables 1 and 2. Information on race or ethnicity was not collected as part of the study and was therefore unavailable for analysis.

### Flow cytometry

PBMCs were isolated from whole blood using density-gradient centrifugation (Histopaque 1077, Merck), cryopreserved in FBS Superior (Biochrom) containing 10% DMSO, and stored at −150°C until further use. Prior to staining, PBMCs were rapidly thawed at 37°C and rested in complete RPMI medium for 20 minutes at 37°C and 5% CO_2_. Splenocytes were obtained by mechanical disruption of spleens followed by RBC lysis using ACK lysis buffer (Thermo Fisher Scientific). For intracellular detection of SMN protein, 1 × 10^6^ cells (PBMCs and splenocytes) were fixed and permeabilized using the FoxP3/Transcription Factor Staining Buffer Set (eBioscience) according to the manufacturer’s instructions. Intracellular staining was performed using purified mouse anti-SMN and donkey anti–mouse IgG (H+L) AF568 or AF647 antibodies. To avoid nonspecific binding of the anti-mouse secondary antibody to surface epitopes, surface marker labeling was performed following intracellular staining. Fc receptors were blocked with human Ig or purified rat anti–mouse CD16 and anti–mouse CD32 (BD Biosciences) antibodies, respectively. Then, cells were labeled with fluorochrome-conjugated monoclonal antibodies against relevant lineage and activation markers. Dead cells were excluded using Zombie NIR fixable live/dead stain (BioLegend). Following surface staining, cells were washed and resuspended in PBS. Spectral flow cytometry was performed using the ID7000 Spectral Cell Analyzer (SONY Biotechnology). Data were analyzed with FlowJo software (version 10.10.0). A complete list of antibodies, including fluorophores, clones, and suppliers, is provided in Supplemental Tables 3–5.

### Statistics

Statistical analysis was performed in GraphPad Prism version 10.4.1 (GraphPad Software). For each immune cell cluster, group differences were analyzed using a 2-tailed Student’s *t* test. A *P* value of less than 0.05 was considered statistically significant, and data are presented as the mean ± SD. Correlation analysis was performed by linear regression with 95% CIs; the coefficient of determination (*r^2^*) and *P* values are indicated in each figure. Human samples were acquired in 2 separate experiments after randomization of controls; SMA samples were matched by age. Differences between treatment groups and treatment effects were assessed using 1-way ANOVA followed by Šidák’s multiple-comparison test. A *P* value of less than 0.05 was considered significant. Exact *P* values are indicated where considered relevant, and data are presented as the mean ± SD.

### Study approval

#### Animal studies.

Mouse breeding and tissue preparations were conducted according to the German Animal Welfare law approved by the Lower Saxony State Office for Consumer Protection and Food Safety (LAVES, reference no. 19/3309).

#### Human studies.

Blood donations were approved by the Ethics Committee of Hannover Medical School under ethical approval number 6269-2012 (adult patients with SMA), 9925_BO_S_2021 (SMA patient child) and 6295-2021 (controls) and University Hospital Essen under ethics approval no. 18-8071-BO (patients with SMA). Written informed consent was obtained from all participants prior to sample collection.

### Data availability

Values for all data points in the main and supplemental figures are provided in the Supporting Data Values file. Raw flow cytometric data used to generate histograms and UMAP plots are available from the corresponding author upon reasonable request.

## Manuscript information

### Author contributions.

IT, KRS, NTD, UK, and PC conceived and designed the study. KRS, IT, FC, TS, and CE acquired and analyzed the data. CE, KRS, and IT designed the flow cytometry panels. BB, IT, NTD, KRS, SP, SI, CM, SN, LIS, ML, and TH collected patient samples. UK, PC, EDS, TG, and SP supervised the study. IT, KR, UK, and PC wrote the manuscript. IT, NTD, and PC secured funding for the study. IT, KR, UK, PC, BB, and TG revised the manuscript. All authors reviewed and approved the final version of the manuscript. Shared first authorship was assigned on the basis of study conceptualization, analysis, and writing of the manuscript.

### AI disclosure.

The authors acknowledge the use of ChatGPT (GPT-5.6, OpenAI) accessed between November 2025 and March 2026, to support language refinement, including grammar, wording, and shortening of the Abstract. All intellectual and scientific aspects of the study, including data generation, analysis, interpretation, and conclusions, were generated independently by the authors. The authors carefully reviewed and edited all content produced with the assistance of these tools and take full responsibility for the accuracy, integrity, and originality of the work.

### Conflict of interest.

SP received honoraria as a speaker and consultant from Biogen GmbH, Roche, Novartis, Cytokinetics Inc., Desitin, Italfarmaco, Amylyx, and Zambon; and grants from DGM e.V., the Federal Ministry of Education and Research, the German Israeli Foundation for Scientific Research and Development, the EU Joint Program for Neurodegenerative Disease Research, and Neurodegenerative Research Inc. PC has received research funding from Roche. SN has received speaker honoraria from Roche and Biogen. SI participated in advisory boards and workshops and received personal fees from Novartis and Roche.

### Funding.

This research was supported by the following:

- SMA Europe grant (Call 11, to IT, NTD, and PC).
- PRACTIS Clinician Scientist Program, funded by Hannover Medical School and DFG (DFG ME 3696/3, to BB).
- SMATHERIA partially supported IT, TS, ND, and PC.

### Present addresses.

CE’s present address is: Kiel University, Kiel, Germany. FC’s present address is: IRCCS Synlab SDN, Naples, Italy.

## Supplementary Material

Supplemental data

Unedited blot and gel images

Supporting data values

## Figures and Tables

**Figure 1:**
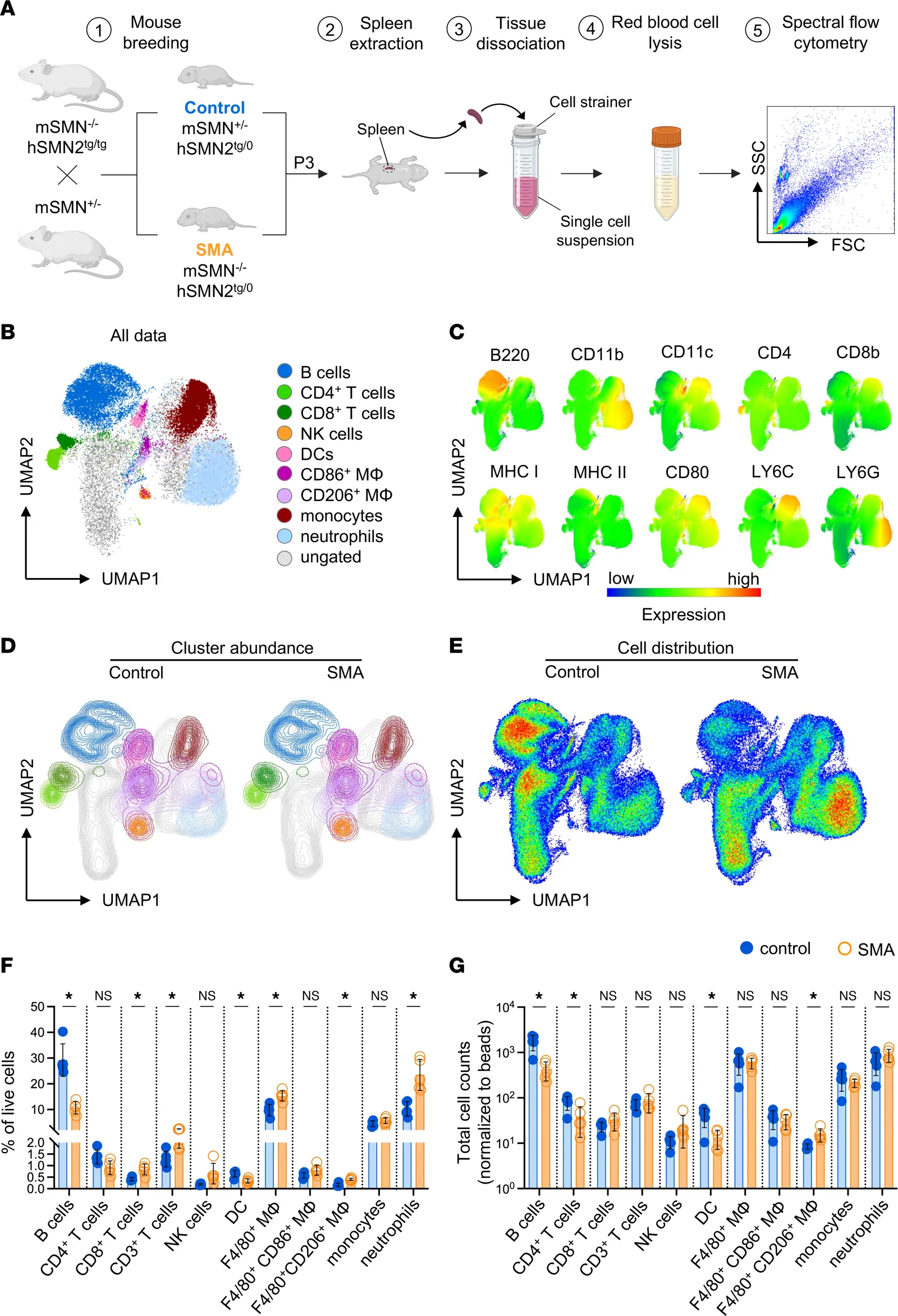
SMA mice show a significant loss of B cells in the spleen at P3. (**A**) Schematic representation of the experimental workflow. Transgenic (*mSmn^–/–^ hSMN2^tg/tg^*) and heterozygous (*mSmn^+/–^*) mice were bred to obtain control (*mSmn^+/–^ hSMN2^tg/0^*) and SMA (*mSmn^-/-^; hSMN2^tg/0^*) offspring. Spleens were extracted at P3 from littermates (SMA *n* = 5, control *n* = 5), followed by tissue dissociation, RBC lysis, and spectral flow cytometric analysis of single-cell suspensions. FSC, forward scatter; SSC, side scatter. (**B**) UMAP representation of splenic immune cells calculated from all samples and phenotypic markers; colored according to cell type. Clustering is based on gating shown in Supplemental Figure 1A. Mφ, macrophages. (**C**) Heatmap-coded UMAPs showing expression intensity of individual surface markers across the identified clusters. (**D**) Contour UMAPs showing immune cell abundance and clustering patterns of splenocytes from SMA and control animals (**E**) Density UMAPs comparing the local distribution and abundance of immune cell populations between control and SMA animal data. (**F** and **G**) Quantification of flow cytometric data for relative abundances (**F**) and total cell counts (**G**) of immune cell subsets in control (blue, filled) and SMA mouse spleens (orange, circle). Data represent the mean ± SD. Each data point is an individual biological replicate. An unpaired, 2-tailed Student’s *t* test without multiple comparisons for each cell type data set was used separately. *P < 0.05.

**Figure 2:**
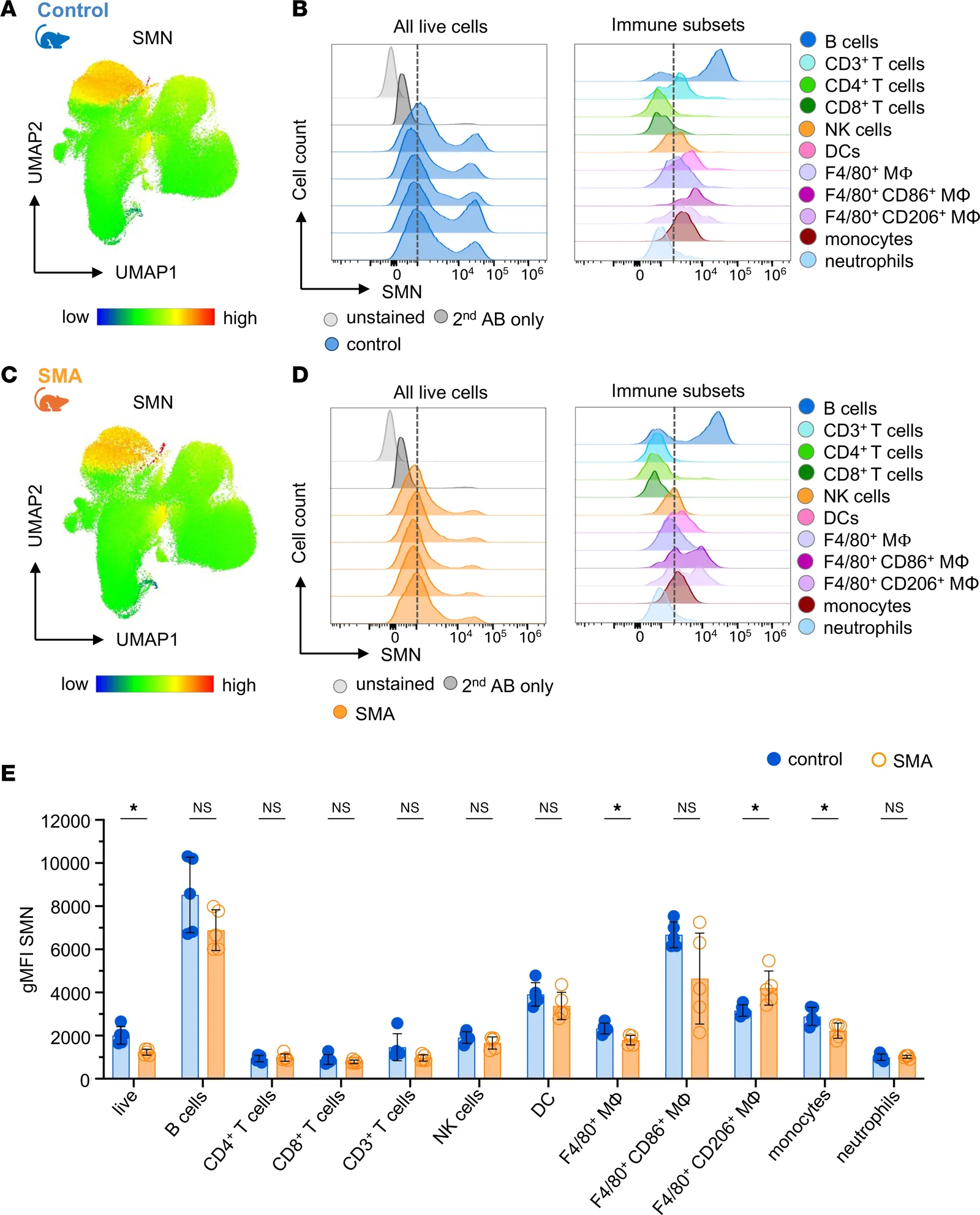
B cells and macrophages express particularly high SMN levels in the spleens of neonatal mice. (**A**) UMAP with heatmap visualization of SMN expression in immune cells from control mice. (**B**) Histogram overlays displayed on a subpanel-specific relative half-offset scale (*y* axis) showing total SMN protein expression (*x* axis) across all immune cells in control mice (*n* = 5, left) and in individual immune cell clusters of 1 representative animal (right), colored according to cluster identity. 2nd AB only, secondary antibody only. (**C**) UMAP visualization of SMN expression in immune cells from SMA mice, using the same heatmap scale as in **A**. (**D**) Histogram overlays displayed as in **B** for SMA mice (*n* = 5, left) and the corresponding immune cell clusters of 1 representative animal (right), matching the cluster identities shown in **B**. Histograms were independently normalized within each subpanel, with the *y* axis representing the percentage of the maximum bin count in that subpanel. Therefore, the histogram heights do not reflect the absolute immune cell subset frequencies and should only be compared within a given subpanel. For comparisons of immune cell frequencies between subpanels, refer to Figure 1. The *x* axis uses a common scale for SMN expression (*x* axis) across all panels. Vertical reference lines indicate identical *x* values for better comparison between control and SMA samples. (**E**) Quantification of gMFI SMN protein expression levels across immune cell clusters in control (blue) and SMA (orange) littermates at P3. Each data point represents an individual biological replicate. Data indicate the mean ± SD. An unpaired, 2-tailed Student’s *t* test without multiple comparisons was used for each cell-type data set. *P < 0.05.

**Figure 3:**
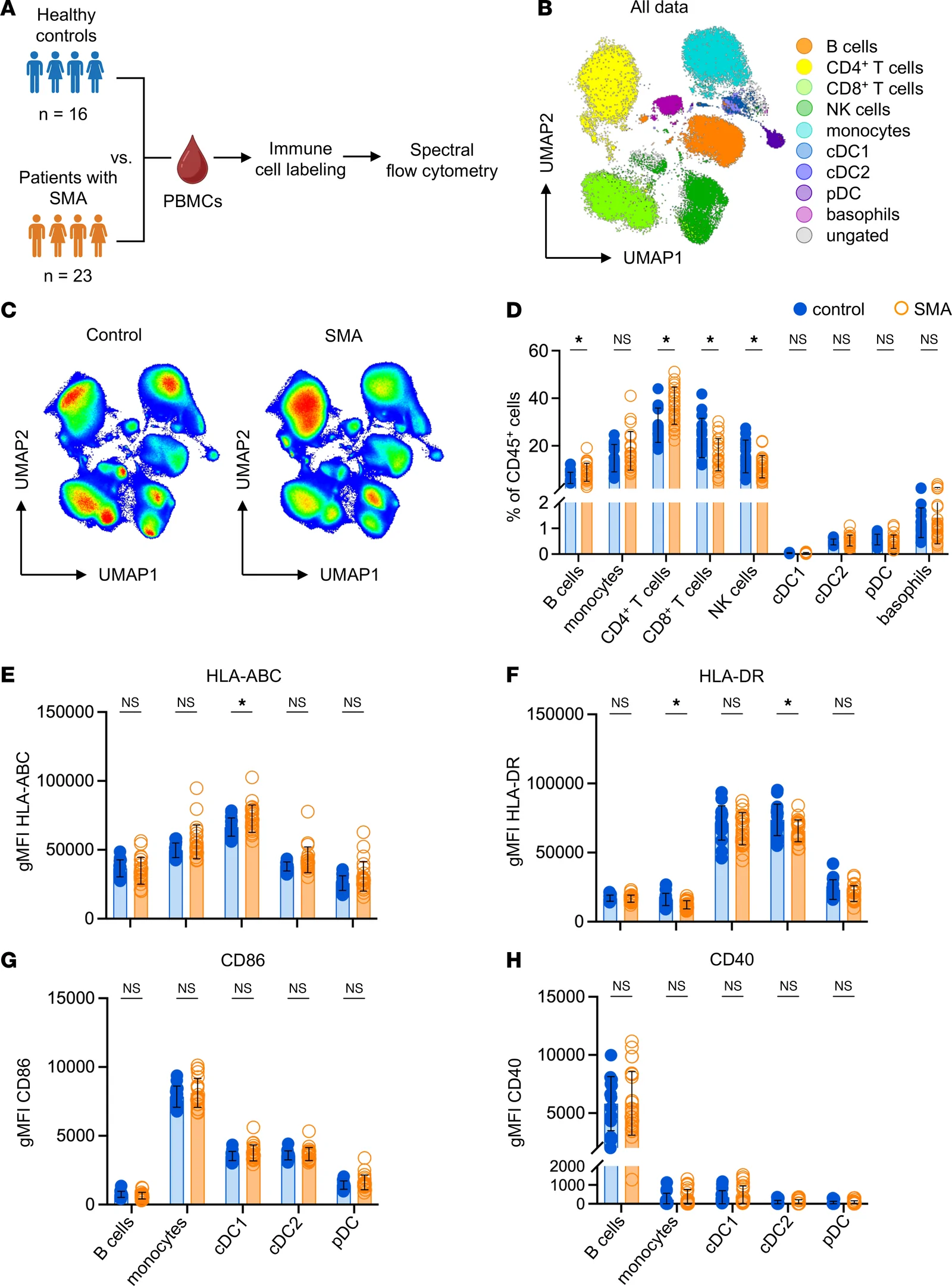
B, T, and NK cell abundances are altered in peripheral blood of treated adult patients with SMA. (**A**) Experimental scheme: PBMCs were isolated from healthy controls (*n* = 16) and patients with SMA (*n* = 23), followed by immune cell labeling and spectral flow cytometric analysis. (**B**) UMAP projection of CD45^+^ immune cells from all study participants based on phenotypic gates, colored according to cluster identity. (**C**) UMAP density plots of CD45^+^ immune cells from controls and patients with SMA, highlighting overall cellular composition. (**D**) Quantification of immune cell subset frequencies as a percentage of CD45^+^ cells in controls (blue) and patients with SMA (orange). Data represent the mean ± SD. *P < 0.05, by unpaired, 2-tailed Student’s *t* test without multiple test correction. (**E**–**H**) Expression levels of activation and antigen presentation markers on the indicated cell types, shown as gMFI: (**E**) HLA-ABC, (**F**) HLA-DR, (**G**) CD86, and (**H**) CD40. Data indicate the mean ± SD. Data represent the mean ± SD. *P < 0.05, by unpaired, 2-tailed Student’s *t* test without multiple test correction.

**Figure 4:**
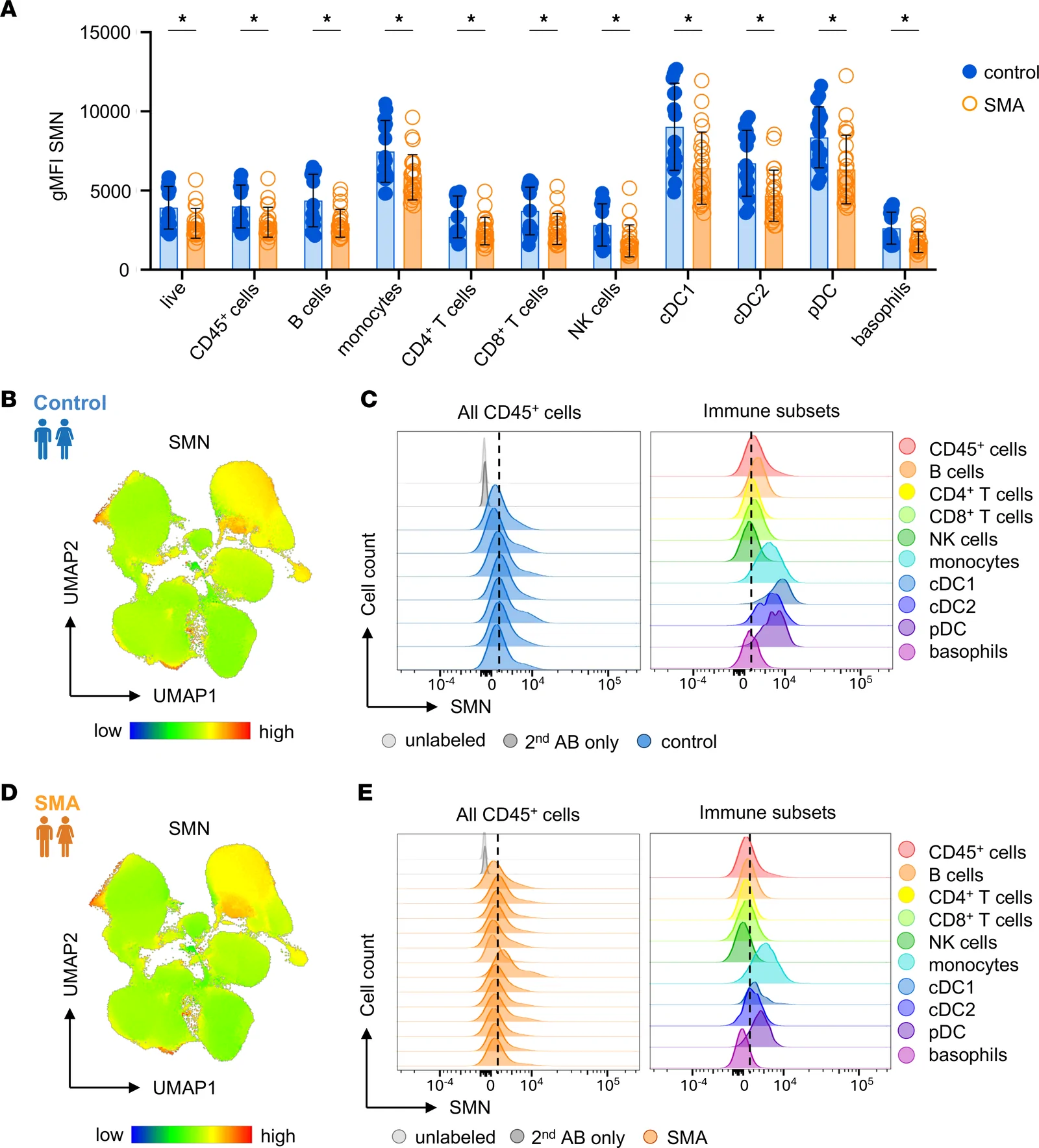
SMN expression is reduced across all immune cell types of treated adult SMA patients. (**A**) Quantification of gMFI SMN protein expression across immune cell clusters in healthy controls and patients with SMA. Each data point represents an individual biological replicate, and data represent the mean ± SD. Statistical comparisons between groups were performed for each cell type using an unpaired, 2-tailed Student’s *t* test without multiple comparisons. *P < 0.05. Samples were acquired in 2 independent experiments performed on consecutive days. Statistical analyses include all samples from both experiments, whereas the histogram overlays and UMAP visualizations shown in **B**–**E** are representative of 1 experiment. (**B**) UMAP visualization of SMN expression in CD45^+^ immune cells from a representative healthy control dataset, displayed using a heatmap color scale. SMN expression is shown as the gMFI. (**C**) Histogram overlays showing total SMN protein expression levels across all immune cells in PBMCs from healthy controls (*n* = 7, left) and in individual immune cell clusters of 1 representative control sample (right), colored according to cluster identity. (**D**) UMAP visualization of SMN expression in immune cells from patients with SMA, using the same heatmap scale as in **B**. (**E**) Histogram overlays showing total SMN protein expression in PBMCs from patients with SMA (*n* = 13, left) and in individual immune cell clusters of 1 representative risdiplam-treated patient, matching the cluster identities shown in **C**. Histograms were normalized independently for each subpanel, with the *y* axis representing the percentage of the maximum bin count within that subpanel (for comparisons of immune cell frequencies between subpanels, refer to Figure 3). The *x* axis shows SMN expression on a common scale across all panels. Vertical reference lines indicate identical *x* values for better comparison between control and SMA samples.

**Figure 5:**
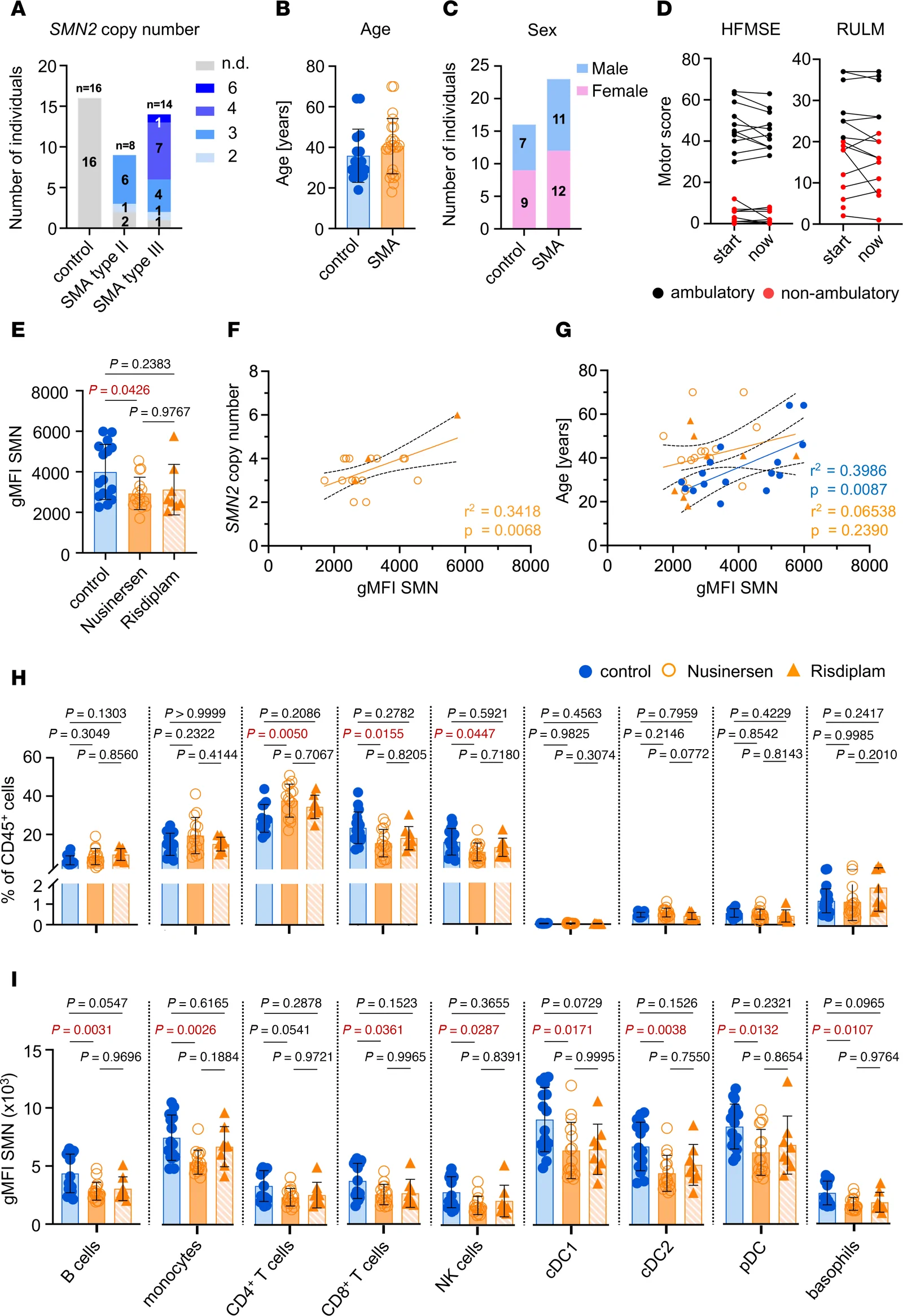
Adult patients with SMA treated with nusinersen exhibit a more pronounced change in PBMC composition compared with those treated with risdiplam. (**A**) Distribution of *SMN2* gene copy numbers among patients with SMA. Controls (gray, *n* = 16) and patients with SMA (type II, *n* = 9; type III, *n* = 14) are shown. The number of *SMN2* copies (copies 2, 3, 4, and 6) is indicated by different shades of blue; n.d., not determined. (**B**) Age distribution for healthy controls (blue) and patients with SMA (orange). Data indicate the mean ± SD. (**C**) Sex distribution of study participants. (**D**) Clinical motor function scores for patients with SMA at initial presentation and at the time of analysis. The HFMSE and RULM scores are shown for ambulatory (black) and nonambulatory (red) individuals. (**E**) SMN expression levels (gMFI) of CD45^+^ in controls (blue), nusinersen-treated patients (*n* = 15, orange, circle), and risdiplam-treated patients (*n* = 8, orange stripes, triangle). One-way ANOVA followed by Šidák’s multiple-comparison test was performed to analyze differences between groups; *P* < 0.05 was considered significant (red); exact *P* values are displayed. (**F** and **G**) Correlation of SMN levels (gMFI) to *SMN2* gene copy number (**F**) and age (**G**) by linear regression with 95% CI; coefficient of determination (*r^2^*) and *P* values are shown. Treatment groups are indicated by data point shape. The legend is shown in **H**. (**H** and **I**) Quantification of immune cell subset frequencies as a percentage of CD45^+^ cells (**H**) and gMFI SMN levels (**I**) in control individuals (blue) and treated patients with SMA (nusinersen – orange + circle, risdiplam – striped orange + triangle). Data represent the mean ± SD. Statistical significance was assessed using 1-way ANOVA followed by Šidák’s multiple-comparison test; *P* < 0.05 was considered significant (red); exact *P* values are displayed.

**Figure 6:**
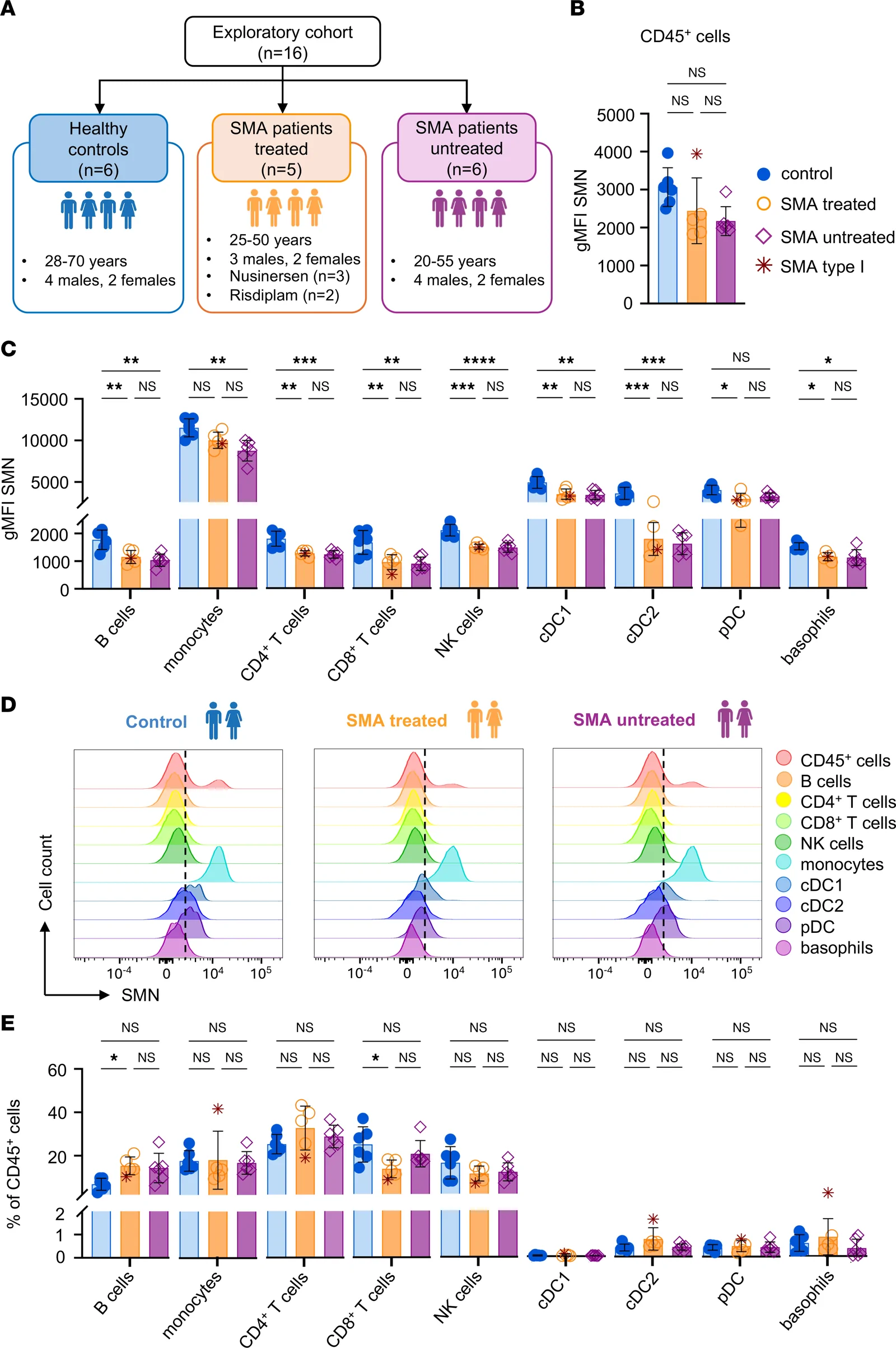
Adult patients with SMA treated with nusinersen or risdiplam show comparable reduction of SMN expression and alterations in PBMC composition relative to untreated patients with SMA. (**A**) Exploratory cohort scheme: analysis of PBMCs from healthy controls (*n* = 6), nusinersen-treated patients with SMA, or risdiplam-treated patients with SMA (a subset of the study cohort above and 1 SMA type I patient, *n* = 5) and untreated patients with SMA (*n* = 6). Clinical data are listed in Supplemental Table 2. (**B** and **C**) SMN expression levels (gMFI) of CD45^+^ cells (**B**) and immune cell subsets (**C**) in controls (blue circles), treated patients (orange circles, type I with red asterisk), and untreated patients (purple diamonds). Data are presented as the mean ± SD. *P < 0.05, **P < 0.01, ***P < 0.001, and ****P < 0.0001, by 1-way ANOVA followed by Šidák’s multiple-comparison test. (**D**) Histogram overlays showing total SMN protein expression levels across immune cell subsets in 1 representative individual per analysis group, colored according to cluster identity. Histograms were normalized independently for each subpanel, with the *y* axis representing the percentage of the maximum bin count within that subpanel. The *x* axis shows SMN expression on a common scale across all panels. Vertical reference lines indicate identical *x* values for better comparison between control, SMA treated, and SMA untreated samples. (**E**) Quantification of immune cell subset frequencies as a percentage of CD45^+^ cells in controls, treated patients with SMA, and untreated patients with SMA. *P < 0.05, by 1-way ANOVA followed by Šidák’s multiple-comparison test.

**Figure 7:**
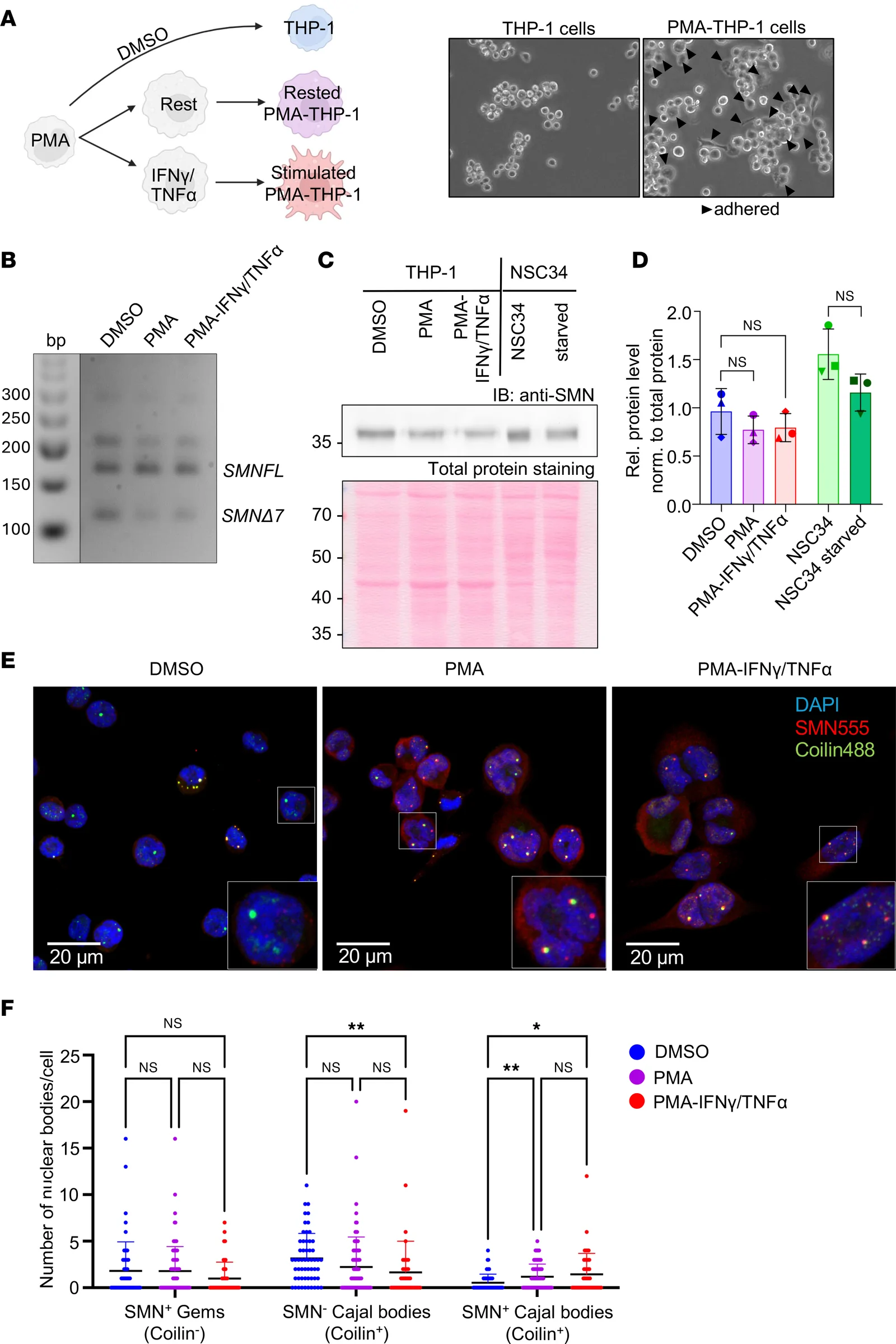
SMNΔ7 mRNA levels decrease and number of SMN^+^ Cajal bodies increase during PMA-induced THP-1 differentiation. (**A**) Schematic depiction of PMA-induced THP-1 differentiation and subsequent IFN-γ and TNF-α stimulation. As a control, cells were treated with DMSO devoid of PMA. The arrows indicate adherent cells. Images were taken in 20x magnification. (**B**) Representative (*n* = 1) agarose gel showing SMN splicing products amplified using primers spanning exons 6–8. (**C**) Western blot analysis of SMN protein levels in THP-1 cells, PMA-stimulated THP-1 cells, and PMA–IFN-γ/TNF-α–stimulated THP-1 cells, and the motoneuron-like cell line NSC34 with and without starvation for cellular differentiation. Data were normalized to total protein by Ponceau S staining. A representative blot for 3 individual cell cultures and blots is shown. (**D**) Quantification of SMN protein levels by Western blotting. A multiple-comparison, 2-tailed Student’s *t* test was performed to determine significance. Data represent the mean ± SD. (**E**) Representative confocal microscopy images analyzing SMN localization (red) in THP-1 cells (*n* = 56), PMA-differentiated THP-1 (PMA–THP-1, *n* = 87), and IFN-γ/TNF-α–stimulated PMA–THP-1 (*n* = 45). Nuclei were stained with DAPI (blue), and Cajal bodies are labeled as Coilin^+^ (green). Scale bar: 20 μm. Enlarged cells are highlighted by a white box and show a digitally magnified region of interest. (**F**) Quantification of SMN^+^ nuclear bodies per cell. Nuclear bodies were categorized as SMN^+^ Gems (Coilin^–^) or SMN^–^ or SMN^+^ Cajal bodies (Coilin^+^). *P < 0.0 and ** *P* < 0.01, by 1-way ANOVA with multiple-comparison correction. Each dot represents a single cell, and data represent the mean ± SD.

## Version 1 08/25/2026

In-Press Preview

## Version 2 11/02/2026

Electronic publication
