# Supplementary material for: Immune cell–specific SMN expression and sustained immune alterations in treated spinal muscular atrophy
Source: J Clin Invest. 2026 Aug 25;136(21):e203540. doi: 10.1172/JCI203540 (PMC13722399; doi:10.1172/JCI203540)
Supplement: Supplementary file 1 [file jci-136-203540-s266.pdf]

SUPPLEMENT

Immune Cell Subset–Specific SMN Expression and Sustained Immune Alterations in Treated Spinal Muscular Atrophy

Ines Tapken, **Katharina Rahmel-Stein**, Christine Ehlers, Federica Cieri, Nora Tula Detering, Tobias Schüning, Bogdan Bjelica, Charlotte Mindermann, Svenja Neuhoff, Linda-Isabell Schmitt, Markus Leo, Tim Hagenacker, Sabine Illsinger, Elia Di Schiavi, Theresa Graalmann, Susanne Petri, Ulrich Kalinke, Peter Claus

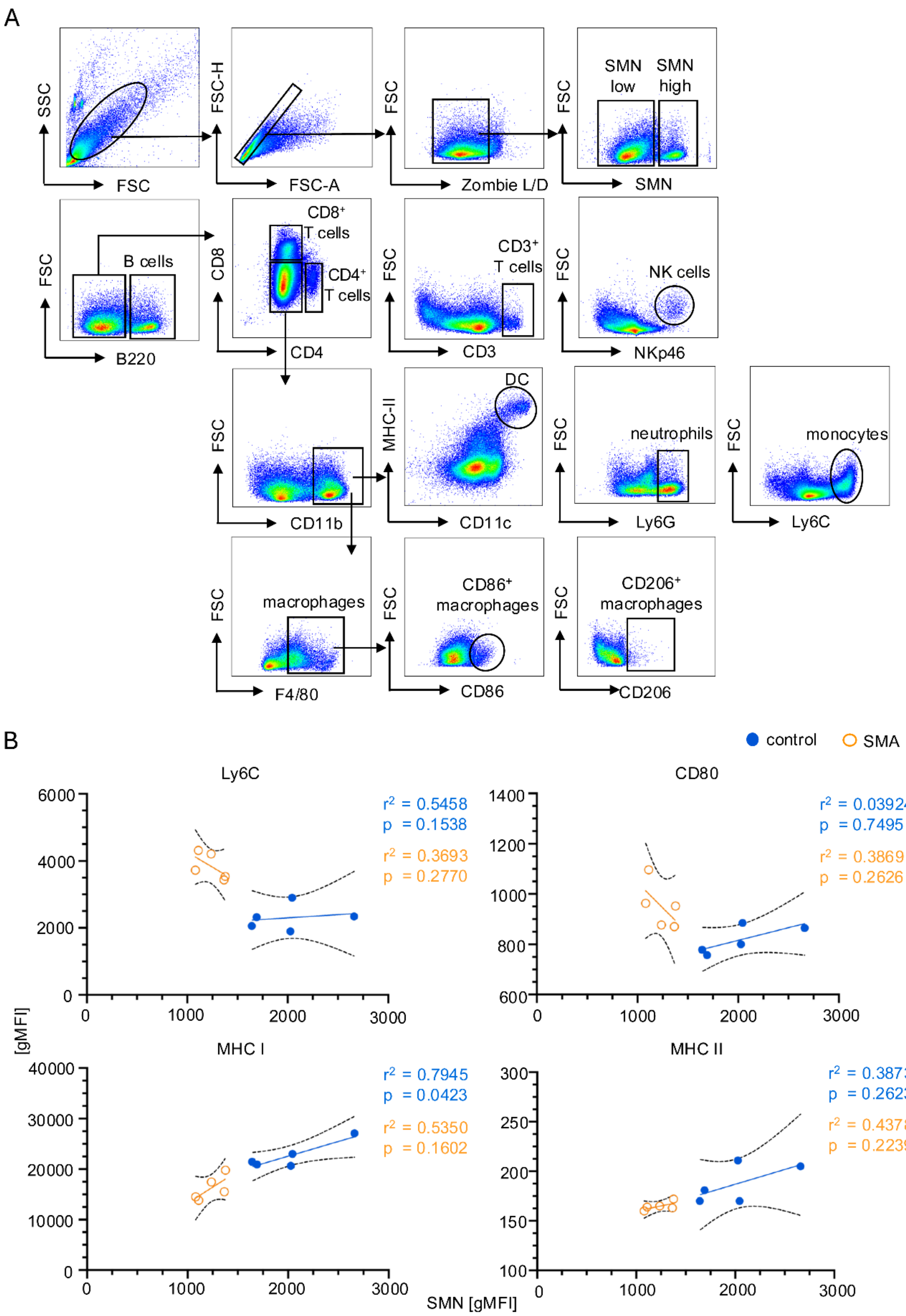

Supplemental Figure 1: (A) Representative flow cytometry gating strategy for the identification of immune cell populations from mouse spleen cells. Sequential gating was applied to select live cells, single cells, followed by discrimination of SMN^{low} and SMN^{high} populations. B cells were gated from live cells as $B220^+$, T cells as $CD3^+$, and subdivided into $CD4^+$ and $CD8^+$ subsets. NK cells were gated as $NKp46^+$, dendritic cells (DC) as $CD11c^+MHC\text{-}II^+$, neutrophils as $Ly6G^+$, and monocytes as $Ly6C^+$. Macrophages were identified as $F4/80^+CD11b^+$ and further classified into $CD86^+$ and $CD206^+$ subsets. **(B)** Correlation analysis of SMN protein expression (gMFI) with differentiation associated surface marker expression (CD80, Ly6C, MHC I, and MHC II; gMFI) from control (blue) and SMA (orange) mice. Each dot represents an individual littermate (orange = SMA (n=5); blue = control (n=5); one litter) Linear regression lines with 95% confidence intervals; coefficient of determination (r^2) and *P* values are indicated.

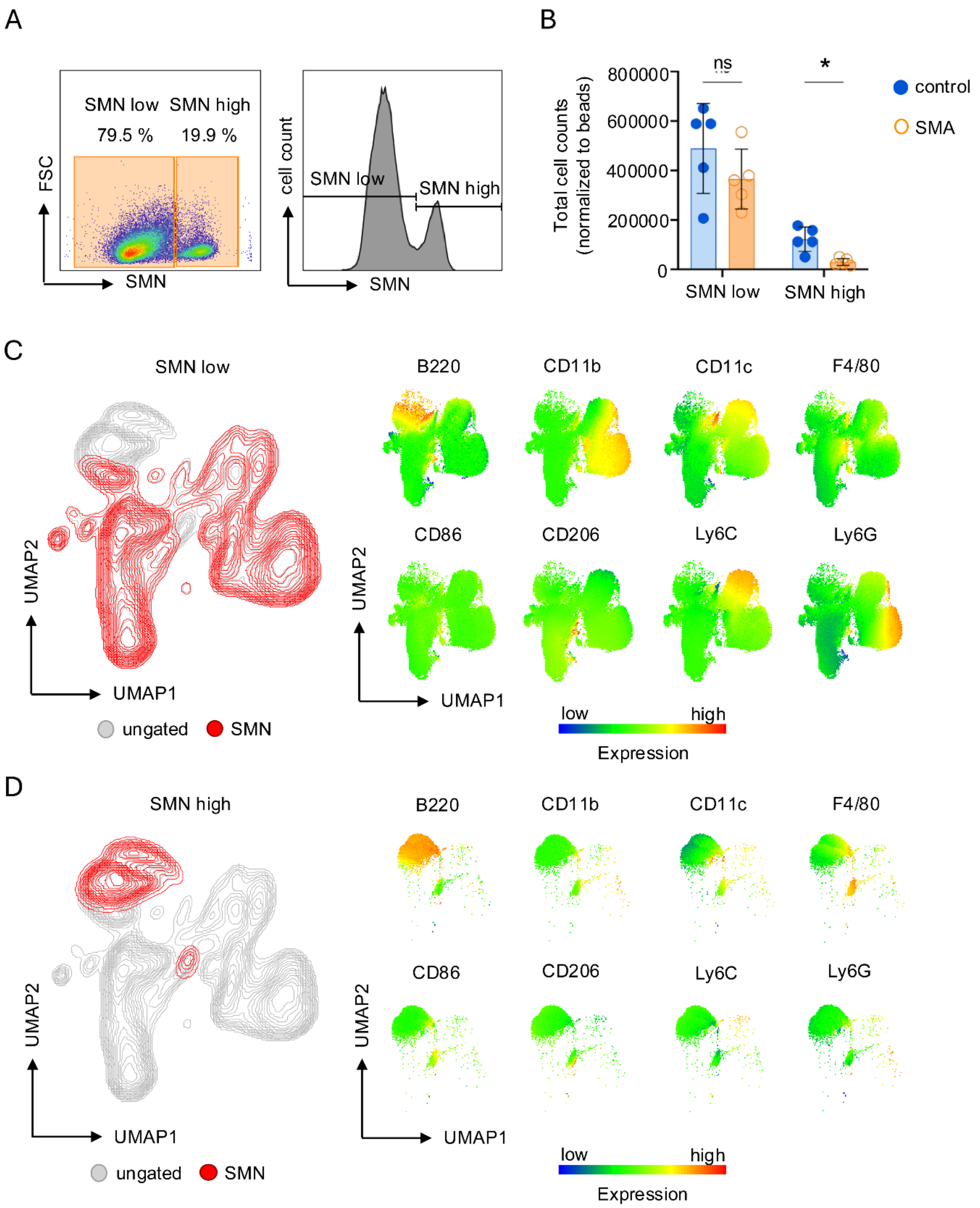

Supplemental Figure 2: (A) Gating strategy for the identification of SMN^{low} and SMN^{high} cell populations based on SMN intensities [gMFI]. Representative dot plot and histogram display the two populations, along with their relative proportions. **(B)** Quantification of the total cell count of SMN^{low} and SMN^{high} cells in control (blue, n=5) and SMA (orange, n=5) mice. Bars indicate mean ± SD. Unpaired T-test **P* < 0.05, ns = not significant. **(C,D)** Density contour plot overlay of SMN^{low} (C) and SMN^{high} (D) populations on UMAP representation of concatenated samples (left). UMAP projections showing expression levels of selected surface markers in SMN^{low} and SMN^{high} cells (right).

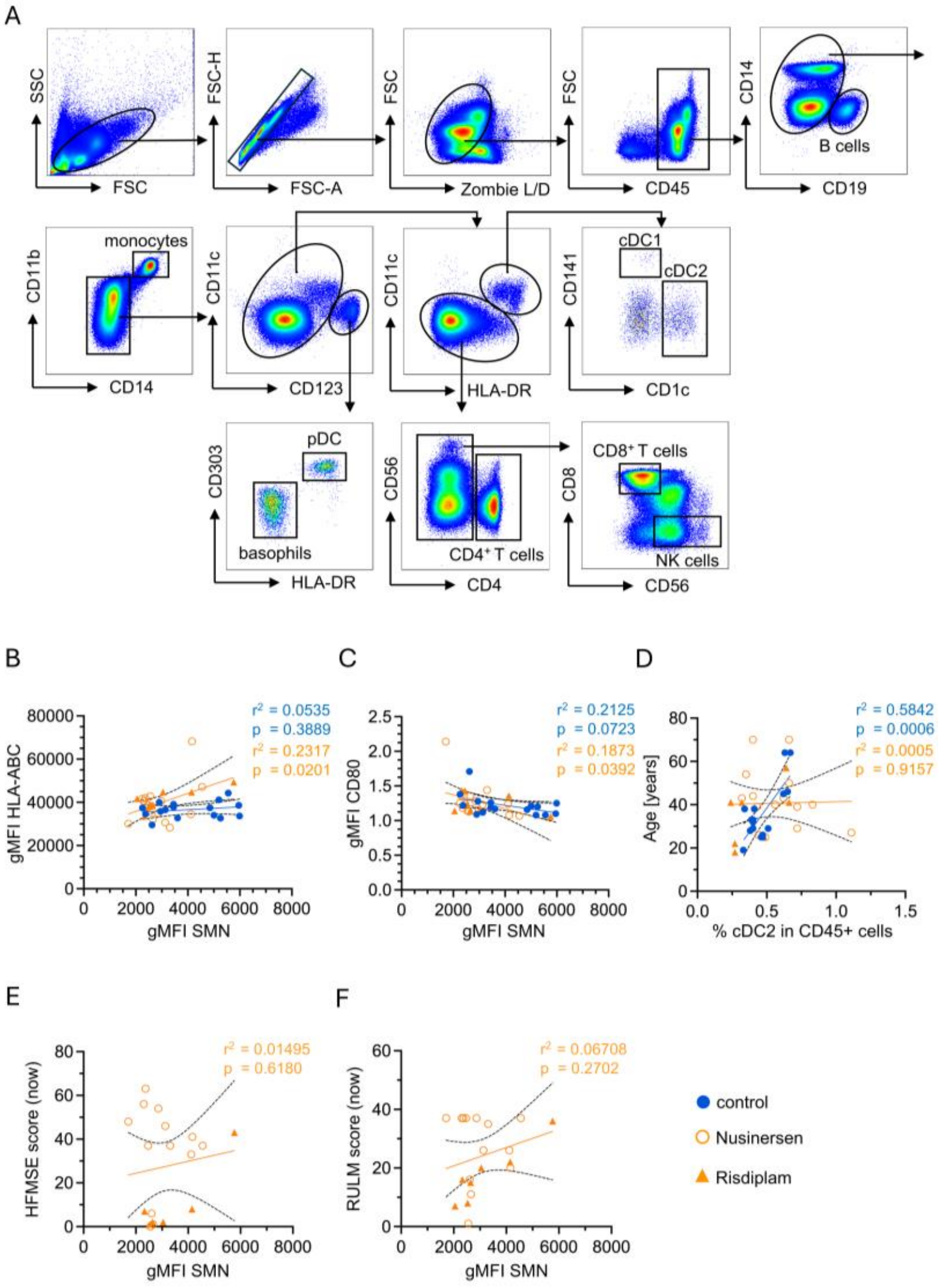

Supplemental Figure 3: (A) Gating strategy of spectral flow cytometry data of PBMC samples from SMA patients (n=23) and healthy controls (n=16). Sequential gating was applied to select live cells, single cells, followed by discrimination of CD45+ leukocytes, CD19+CD14- B cells, CD14+ monocytes, CD123+CD303-HLA-DR- basophils, CD123+CD303-HLA-DR- pDCs, CD11c+HLA-DR+ cells were further subdivided into cDC1 and cDC2 subgroups. CD11c- HLA-DR- cells were further gated as CD4+ T cells, CD8+ T cells and CD56+ NK cells. **(B-C)** Correlation of SMN levels [gMFI] to HLA-ABC and CD80 [gMFI] by linear regression with 95% confidence interval, coefficient of determination (r^2) and *P* values are indicated. Treatment subgroups are indicated by data point shape (SMA orange, Nusinersen circle, Risdiplam triangle, control blue dot). **(D)** Correlation of frequency [%] of cDC2 in CD45+ cells to patients age at time of blood draw. Statistics are equal to B. **(E-F)** Correlation of SMN levels [gMFI] to motor scores (HFMSE, RULM). Statistics are equal to B.

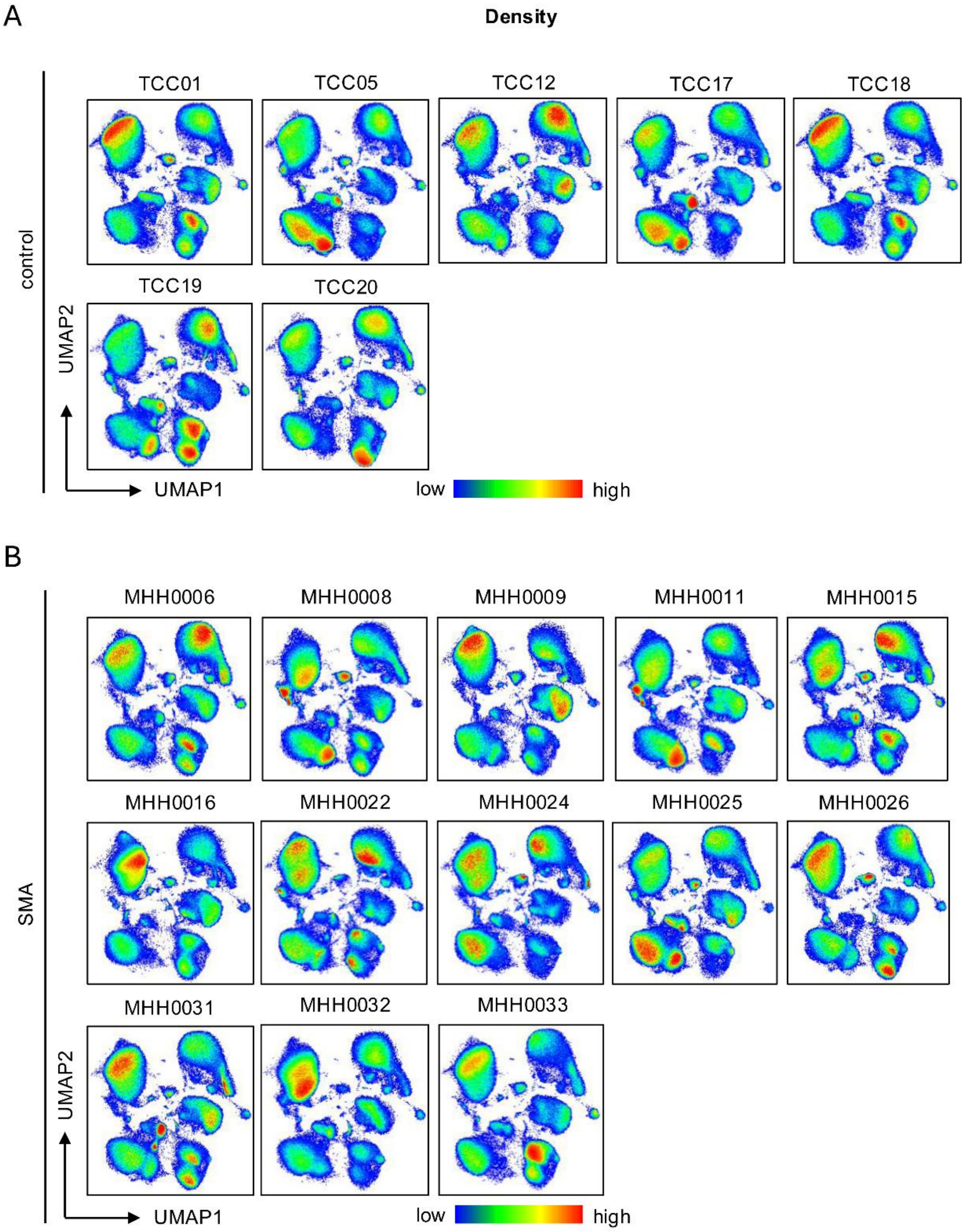

Supplemental Figure 4: (A) Healthy control (TCC numbers) and **(B)** SMA patient (MHH numbers) individual UMAP density plots of CD45^{+} immune cells, highlighting overall cellular composition.

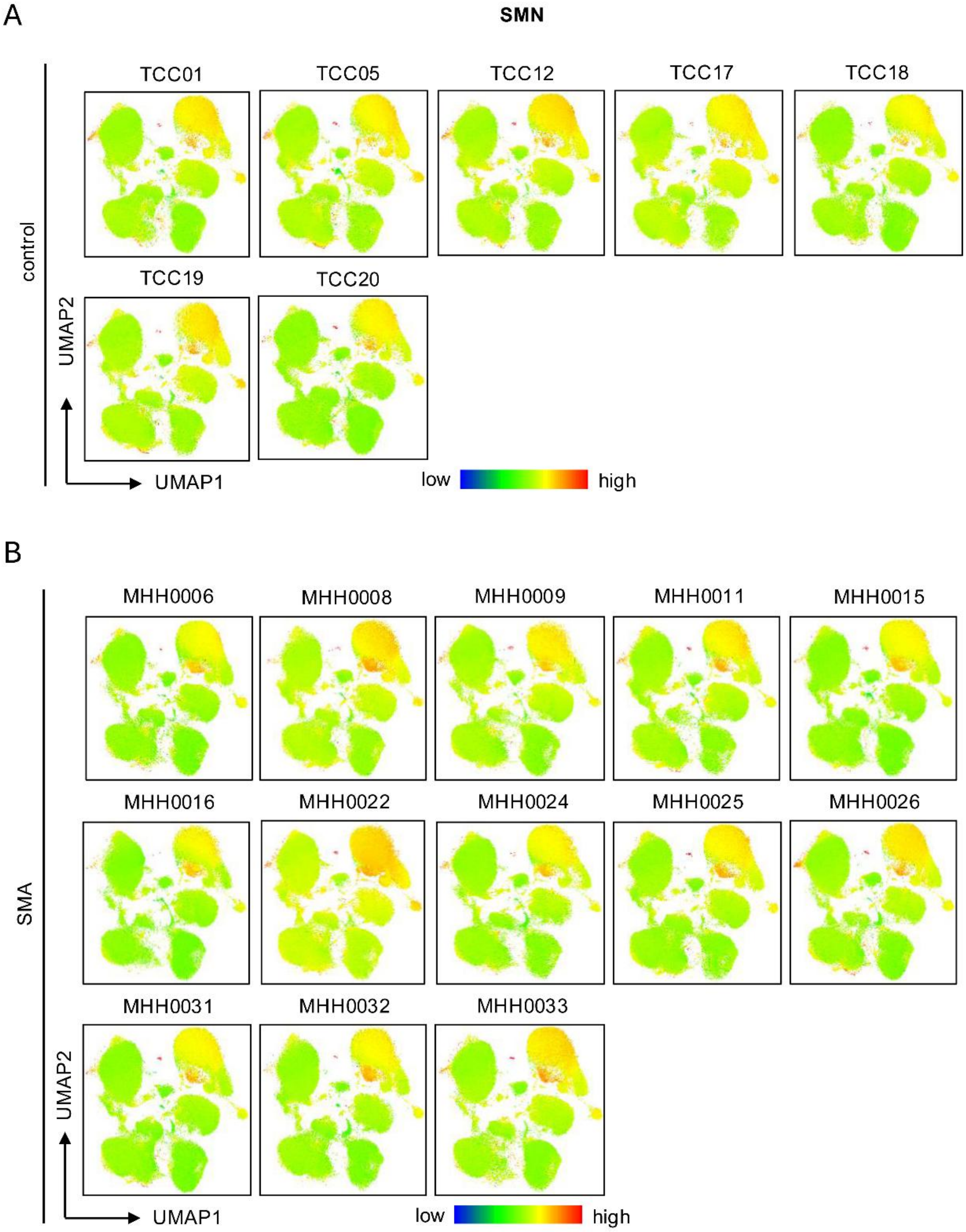

Supplemental Figure 5: (A) Healthy control (TCC numbers) and **(B)** SMA patient (MHH numbers) individual UMAP density plots of CD45⁺ immune cells, highlighting SMN expression.

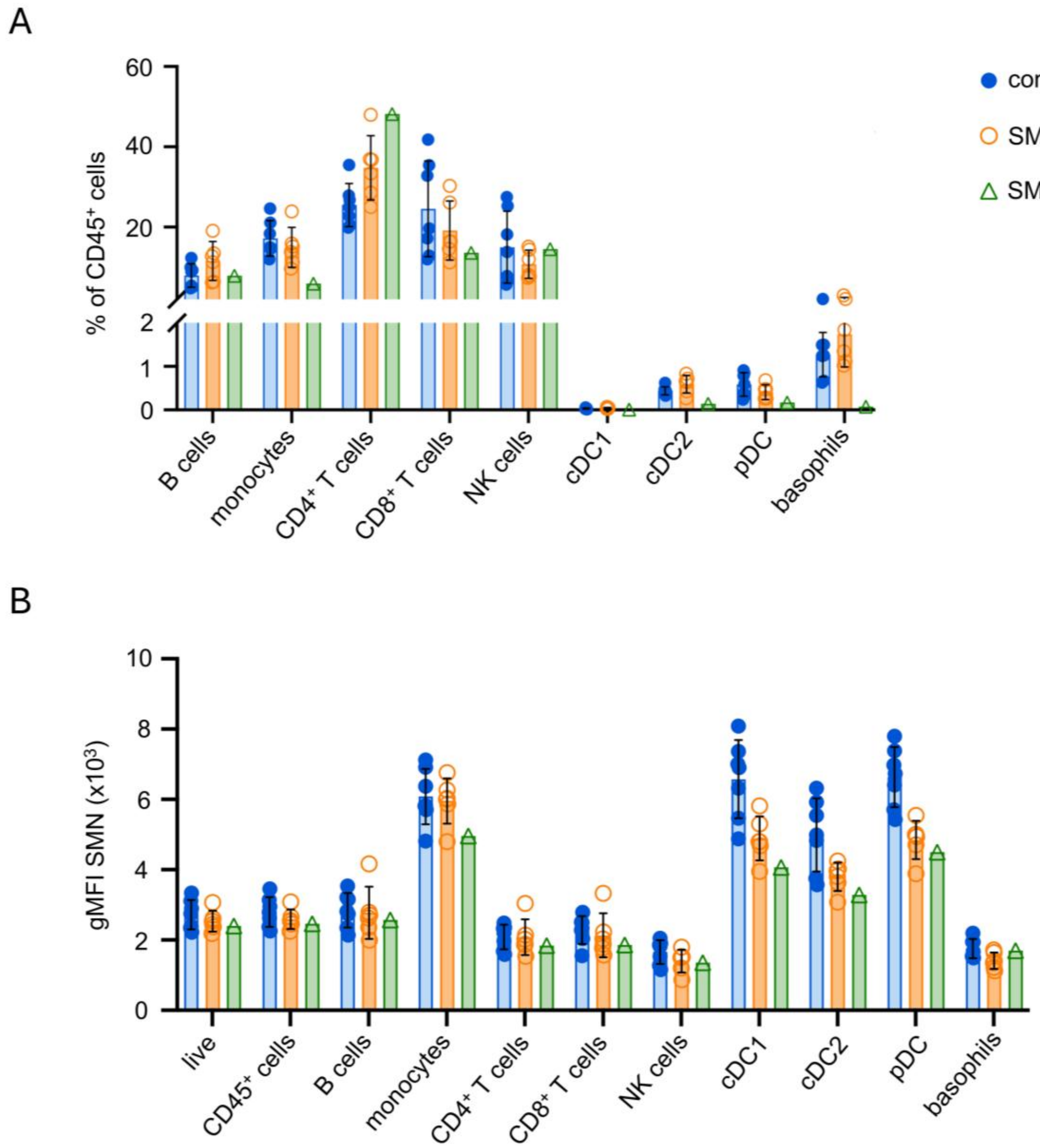

Supplemental Figure 6: Descriptive data (n=1): Monocyte frequency in PBMCs from one infant SMA patient is decreased compared to adult SMA patients and showes lower SMN levels. (A) Immune cell distribution of CD45+ cells in PBMCs from an untreated infant (n=1, präsymptomatic SMA, 3 copies of *SMN2*, green triangle, adult (n=5, orange circle) SMA patients treated with Nusinersen matched by copy number (3 *SMN2* copies) and control adults matched by age (n=7, blue dot). **(B)** SMN levels [gMFI] from immune cell populations as shown in A including CD45+ and live cells.

Supplemental Table 1: Clinical characteristics of main study of SMA patients and healthy controls at time of blood draw

**Characteristic**	**Treated SMA (n=23)**	**Control (n=16)**
Mean age y (range)	42.3 (18-70)	35.9 (19-64)
Male n (%)	11 (47.8)	7 (43.8)
Female n (%)	12 (52.2)	9 (56.3)
**Treatment type**	**n (%)**	
Nusinersen (N)	15 (65.2)	-
Risdiplam (R)	8 (34.8)	-
treatment switch (N to R)	3	-
**SMA type**	**n (%)**	
n.d.	1 (4.3)	-
Type II	8 (34.8)	-
Type III	14 (60.9)	-
***SMN1* mutation**	**n (%)**	
n.d.	7 (30.4)	-
Homozygous deletion of exon 7	5 (21.7)	-
Homozygous deletion of exon 7 and 8	6 (26.1)	-
Homozygous deletion in *SMN1*	5 (21.7)	-
***SMN2* copy number**	**n (%)**	
n.d.	3 (13)	-
2	2 (8.7)	-
3	9 (39.1)	-
3 (4)	1 (4.3)	-
4	7 (30.4)	-
6	1 (4.3)	-
**Ambulatory status**	**n (%)**	
non-ambulatory	11 (47.8)	-
ambulatory	12 (52.2)	-
	**average (±)**	
HFMSE (time of analysis)	24.9 (±38/25)	-
RULM (time of analysis)	21.9 (±15/22)	-
HFMSE (first assesment)	24.5 (±39/24)	-
RULM (first assesment)	21.7 (±15/22)	-
**Duration**	**average (±)**	
disease (time of analysis)	34.5 (35/23)	-
treatment (time of analysis)	5.9 (2/4)	-
**Immune status at time of analysis**	**n (comment)**	
infection (reported acute or recently resolved infection occurring within 4 weeks)	23 (no infection at sampling)	-
vaccination (not systematically recorded; since 2020; no abnormalities in response known)	16 (influenza vaccine)<br>2 (Covid-19 vaccine)<br>1 (pneumococci vaccine)<br>6 (n.d.)	-<br>-<br>-<br>-
Autoantibodies measured	19 (normal)	-

Supplemental Table 2: Clinical characteristics of exploratory data from SMA patients and healthy controls at time of blood draw

Characteristic	Treated SMA (n=5)	Untreated SMA (n=6)	Control (n=6)
Mean age y (range)	39 (25-50)	36 (20-55)	47 (28-70)
Male n (%)	3	4	4
Female n (%)	2	2	2
**Treatment type**	**n**	**n**	
Nusinersen (N)	3	-	-
Risdiplam (R)	2	-	-
**SMA type**	**n**	**n**	
Type I	1	-	-
Type II		3	-
Type III	3	3	-
n.d.	1	-	-
***SMN1* mutation**		**n**	
Homozygous deletion of exon 7 and 8	-	5	-
Homozygous deletion in *SMN1*	2	1	-
n.d.	3	-	-
***SMN2* copy number**		**n**	
2-3	1	-	-
3	1	4	-
3-4	1	-	-
4	1	2	-
n.d.	1	-	-
**Ambulatory status**		**n**	
sitter	1	3	-
non-sitter	-	1	-
non-ambulatory	2	-	-
walker	2	1	-
	**average (±)**	**average (±)**	
HFMSE (first assesment)	24 (±24/23)	14.7 (±41/15)	-
RULM (first assesment)	32 (±16/18)	17.3 (±20/17)	-
**Disease (years)**	**average (±)**	**average (±)**	
age at symptom start	8(±30.8/8)	4.1 (±14/4)	-
Disease duration (time of analysis)	31.4 (±17.9/20.5)	31.5 (±23/22)	-
treatment duration	4.6 (±2.9/2.6)		

Supplemental Table 3: Antibody panel for human PBMC characterization by flow cytometry

Marker	Clone	Fluorophore	Host	Isotype	Cat#	Supplier
Surface staining						
CD14	M5E2	BUV563	Mouse	IgG2a, κ	741360	BD Bioscience
CD19	HIB19	BUV805	Mouse	IgG1, κ	568331	BD Bioscience
CD69	FN50	BUV737	Mouse	IgG1, κ	612818	BD Bioscience
CD80	L307.4	BUV395	Mouse	IgG1, κ	565210	BD Bioscience
CD86	2331	BUV661	Mouse	IgG1, κ	741629	BD Bioscience
CD1c	L161	BV605	Mouse	IgG2b, κ	331538	BioLegend
CD11b	ICRF44	BV570	Mouse	IgG1, κ	301325	BioLegend
CD11c	3.9	BV510	Mouse	IgG1, κ	301634	BioLegend
CD56	MEM-188	PacBlue	Mouse	IgG2a, κ	304629	BioLegend
CD141	1A4	BV711	Mouse	IgG1, κ	563155	BD Bioscience
CD8	SK1	Spark Blue 550	Mouse	IgG1, κ	344760	BioLegend
CD16	3G8	PerCP-Cy5.5	Mouse	IgG1, κ	980118	BioLegend
HLA-ABC	W6/32	PerCP	Mouse	IgG2a, κ	311422	BioLegend
CD40	5C3	PE-Dazzle 594	Mouse	IgG1, κ	334342	BioLegend
CD45	HI30	PE-Cy5.5	Mouse	IgG1	35-0459-42	Invitrogen
CD123	6H6	PE-Cy5	Mouse	IgG1, κ	306008	BioLegend
CD303	201A	PE-Cy7	Mouse	IgG2a, κ	354214	BioLegend
CD3	SK7	Spark NIR 685	Mouse	IgG1, κ	344862	BioLegend
CD4	SK3	APC	Mouse	IgG1, κ	344614	BioLegend
HLA-DR	L243	APC-Cy7	Mouse	IgG2a, κ	307618	BioLegend
Intracellular staining						
SMN	8/SMN	-	Mouse	IgG1	610647	BD Bioscience

Supplemental Table 4: Antibody panel for murine spleen cell characterization by flow cytometry

Marker	Clone	Fluorophore	Host	Isotype	Cat#	Supplier
Surface staining						
MHC-I	M1/42	BUV661	Rat	IgG2a, κ	749702	BD Bioscience
CD80	16-10A1	BUV737	Hamster	IgG2, κ	612773	BD Bioscience
CD45.2	104	BV480	Mouse	IgG2a, κ	566077	BD Bioscience
CD206	C068C2	BV421	Rat	IgG2a, κ	141717	BioLegend
CD4	RM4-5	BV570	Rat	IgG2a, κ	100542	BioLegend
CD86	GL1	BV605	Rat	IgG2a, κ	105037	BioLegend
Ly6G	1A8	BV650	Rat	IgG2a, κ	127641	BioLegend
CD8b	YTS156.7.7	BV711	Rat	IgG2b, κ	126633	BioLegend
B220	RA3-6B2	BV750	Rat	IgG2a, κ	103261	BioLegend
MHC-II	M5/114.15.2	FITC	Rat	IgG2b, κ	107606	BioLegend
Ly6C	HK1.4	PerCP-Cy5.5	Rat	IgG2c, κ	128012	BioLegend
CD11c	N418	PE-Cy5	Hamster	IgG	117318	BioLegend
CD169	3D6.112	PE-Cy7	Rat	IgG2a, κ	142410	BioLegend
F4/80	BM8	PE-Dazzle 594	Rat	IgG2a, κ	123112	BioLegend
CD3	17A2	AF700	Rat	IgG2b, κ	100216	BioLegend
CD11b	M1/70	APC-Cy7	Rat	IgG2b, κ	561039	BD Bioscience
Intracellular staining						
SMN	8/SMN	-	Mouse	IgG1	610647	BD Bioscience

Supplemental Table 5: Secondary antibodies used to counterstain SMN mouse IgG1 in flow cytometry analysis

Target Species	Host Species	Fluorophore	Isotype	Cat#	Supplier
Anti-Mouse	Donkey	Alexa Fluor 568	IgG (H+L)	A10037	Thermo Fisher
Anti-Mouse	Donkey	Alexa Fluor 647	IgG (H+L)	A-31571	Thermo Fisher
