# Supplementary material for: Immune cell–specific SMN expression and sustained immune alterations in treated spinal muscular atrophy
Source: J Clin Invest. 2026 Aug 25;136(21):e203540. doi: 10.1172/JCI203540 (PMC13722399; doi:10.1172/JCI203540)

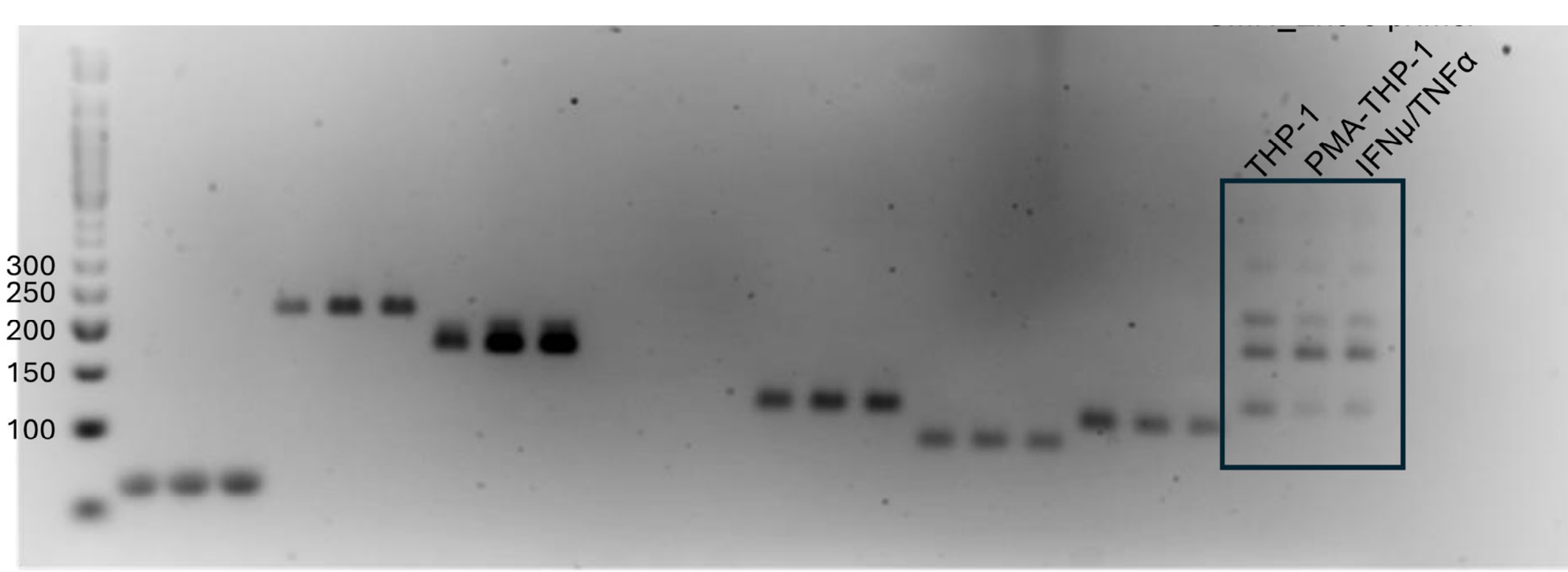

Full unedited blot for Figure 7C

Anti-SMN immunblot

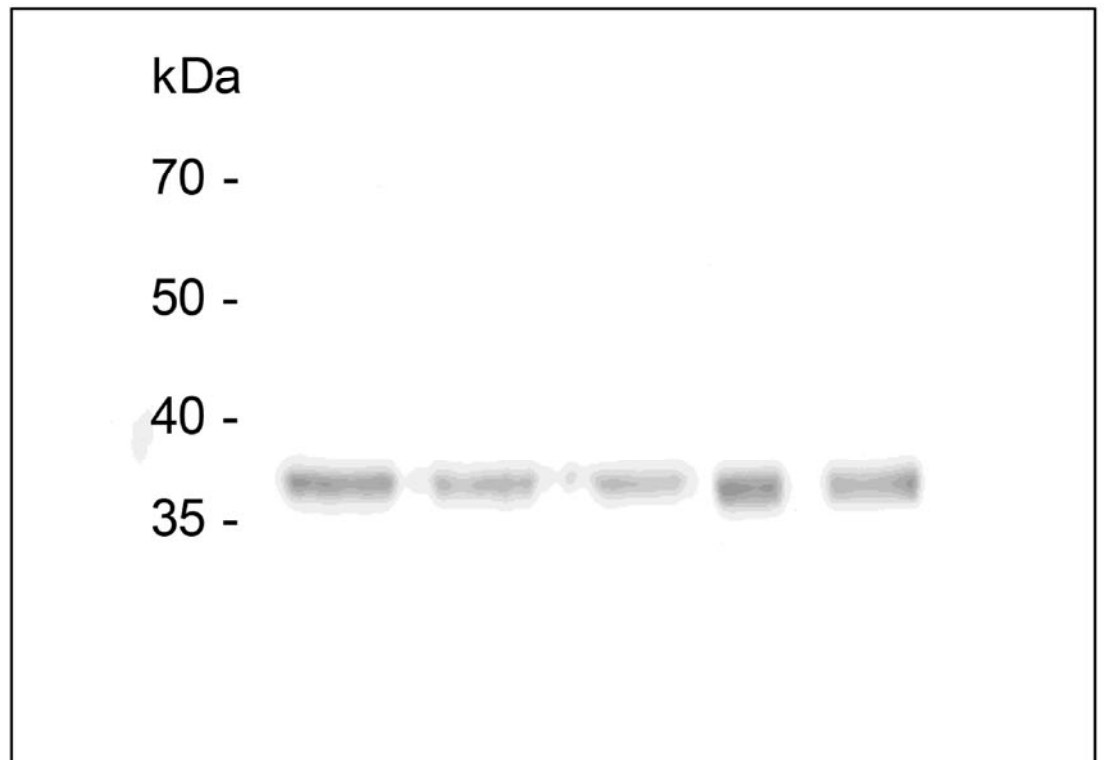

Total protein staining

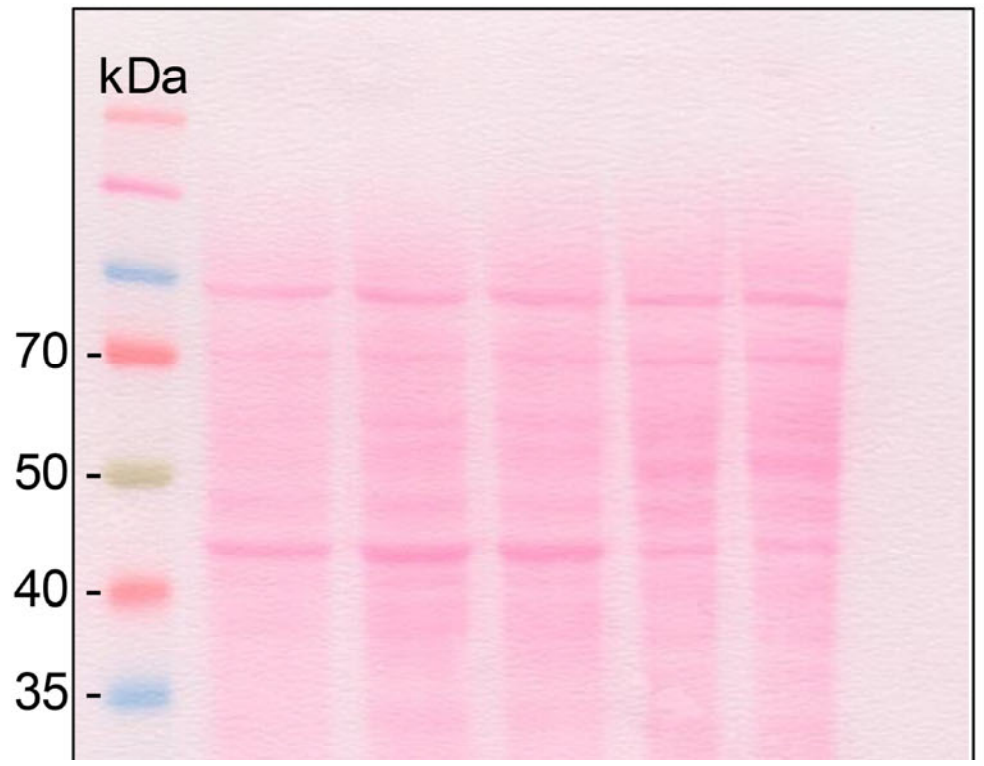

Supplement: Supplementary file 2 [file jci-136-203540-s267.pdf]
